# Non-Chemical Stressors and Cumulative Risk Assessment: An Overview of Current Initiatives and Potential Air Pollutant Interactions

**DOI:** 10.3390/ijerph8062020

**Published:** 2011-06-08

**Authors:** Ari S. Lewis, Sonja N. Sax, Susan C. Wason, Sharan L. Campleman

**Affiliations:** 1 Gradient, 20 University Road, Cambridge, MA 02138, USA; E-Mail: ssax@gradientcorp.com; 2 Department of Environmental Health, Harvard School of Public Health, 401 Park Drive, Boston, MA 02215, USA; E-Mail: swason@hsph.harvard.edu; 3 Electric Power Research Institute, 3420 Hillview Ave., Palo Alto, CA 94304, USA; E-Mail: scampleman@epri.com

**Keywords:** cumulative risk assessment, vulnerable populations, socioeconomic status, social stress, air pollutants

## Abstract

Regulatory agencies are under increased pressure to consider broader public health concerns that extend to multiple pollutant exposures, multiple exposure pathways, and vulnerable populations. Specifically, cumulative risk assessment initiatives have stressed the importance of considering both chemical and non-chemical stressors, such as socioeconomic status (SES) and related psychosocial stress, in evaluating health risks. The integration of non-chemical stressors into a cumulative risk assessment framework has been largely driven by evidence of health disparities across different segments of society that may also bear a disproportionate risk from chemical exposures. This review will discuss current efforts to advance the field of cumulative risk assessment, highlighting some of the major challenges, discussed within the construct of the traditional risk assessment paradigm. Additionally, we present a summary of studies of potential interactions between social stressors and air pollutants on health as an example of current research that supports the incorporation of non-chemical stressors into risk assessment. The results from these studies, while suggestive of possible interactions, are mixed and hindered by inconsistent application of social stress indicators. Overall, while there have been significant advances, further developments across all of the risk assessment stages (*i.e.*, hazard identification, exposure assessment, dose-response, and risk characterization) are necessary to provide a scientific basis for regulatory actions and effective community interventions, particularly when considering non-chemical stressors. A better understanding of the biological underpinnings of social stress on disease and implications for chemical-based dose-response relationships is needed. Furthermore, when considering non-chemical stressors, an appropriate metric, or series of metrics, for risk characterization is also needed. Cumulative risk assessment research will benefit from coordination of information from several different scientific disciplines, including, for example, toxicology, epidemiology, nutrition, neurotoxicology, and the social sciences.

## 1. Introduction

Cumulative risk assessment has existed in some form for many years, such as in the consideration of multiple chemical exposures, sensitive sub-populations, and multi-pathway evaluations in Superfund risk assessments [1]. Cumulative risk assessment, however, has only recently emerged as an area of interest among regulators and stakeholders concerned about environmental justice as a strategy for assessing health impacts in underserved communities [2–5]. As a result, the focus of risk assessment is shifting from assessing hypothetical risks to individual high-impact receptors to assessing community-wide population risks. Concurrently, risk assessment as a science is also undergoing methodological changes. The National Research Council’s (NRC) final “*Science and Decisions*” report, released in 2009, recommended several paradigm shifts for advancing risk assessment, including the need to characterize the effects of multiple stressors, both chemical and non-chemical, on public health [6]. Specifically, the recommendation was to include all chemical, biological, physical, and social stressors in cumulative risk efforts.

Despite the inclusion of non-chemical stressors in the definition of cumulative risk, cumulative risk assessments to date have not included these stressors in a quantitative manner [6,7]. This is largely because few traditional toxicological studies are available to support risk evaluations that consider the combined effects of chemical and non-chemical stressors, and suitable epidemiological information is limited, as summarized in Section 5.2. Additionally, a wealth of information from other disciplines (e.g., psychology, sociology) has yet to be fully integrated into the evaluation of the interactions between chemical and non-chemical stressors and cumulative risk methods for incorporating these data are only now being considered.

Researchers have identified disparities for numerous health outcomes among disadvantaged populations and hypothesize that exposures to combinations of non-chemical and chemical stressors contribute to these disparities (e.g., cancer, asthma, kidney disease, cardiovascular disease, *etc.* [8–12]), but scientists have an incomplete understanding of the mechanisms by which non-chemical stressors alone, or in combination with chemical exposures, contribute to poor health. There is a particular need to determine whether different chemical and non-chemical stressors share a common biological pathway and/or how multiple stressors may modify a chemical dose-response relationship [13]. A critical piece in understanding and incorporating the human health risk impacts from non-chemical stressors is the development of an appropriate exposure or dose metric, or series of metrics, for evaluating these stressors quantitatively.

Current cumulative risk assessment/impact programs are being developed, generally focusing on identifying populations that may have both increased chemical exposure and vulnerability based on a combination of living conditions or social behaviors (e.g., Cumulative Impacts programs in California and New Jersey). For example, several state agencies have developed a new screening methodology for identifying areas potentially affected by cumulative chemical and non-chemical impacts, but the methodology does not serve as a “quantitative assessment of community health impacts, rather it can be used as a relative ranking to distinguish higher-impacted communities from lower-impacted communities and to identify which factors are the greatest contributors to cumulative impact” [2]. Thus, there is a need to develop refined methodologies that can operate within a larger framework of risk-based decision making, and to use existing tools (either qualitatively or quantitatively) to evaluate the impacts of these stressors. As suggested by NRC, the effectiveness of cumulative risk assessments may also be improved by considering what the possible risk management options could be for reducing the hazard or exposure (e.g., the feasibility of regulation, remediation, education, or other interventions) in the scoping and planning phase of the risk assessment, rather than at the end, as is traditionally done [6].

This review will briefly summarize how cumulative risks are currently addressed and discuss current efforts to advance the field of cumulative risk assessment, highlighting some of the major challenges, particularly with respect to inclusion of non-chemical stressors. We will examine the importance of considering the biological mechanism(s) underlying associations between chemical and non-chemical stressors and health, and efforts to include this information in risk evaluations. The discussion will focus on the potential interactions that may occur between chemical and non-chemical stressors and their influence on health outcomes. These concepts are discussed within the construct of the traditional risk assessment paradigm and applied to the evaluation of both individual and community risks. This review is not meant to be an exhaustive analysis of all cumulative risk assessment efforts, but rather a general overview of key initiatives and research needs. As part of our review, and to highlight the type of information that will be necessary to advance the incorporation of non-chemical stressors into risk assessment, we summarize available epidemiological research that explores the interactions between non-chemical stressors and air pollutants. Although results from these studies are mixed, this research provides important insights related to a better understanding of the cumulative impacts of these stressors. We discuss the findings from these studies as well as their limitations.

## 2. What Is Cumulative Risk Assessment?

As risk science evolves, the United States Environmental Protection Agency (US EPA) has been asked to consider broader public health concerns that extend to multiple pollutant exposures, multiple exposure pathways, complex mixtures, and vulnerable population groups. In 2003, US EPA issued guidance on cumulative risk assessment to formalize this more inclusive approach to risk assessment. The cumulative risk assessment framework presented by US EPA [14] defined cumulative risk as “an analysis, characterization, and possible quantification of the combined risks to health or the environment from multiple agents or stressors.” Importantly, US EPA [14] explicitly underscores the need for considering both chemical and non-chemical stressors, with the latter including (and extending beyond) low income, depressed community property values, limited access to health care, psychosocial stress, and other stressors not commonly within the purview of US EPA’s regulatory framework.

Furthermore, US EPA’s framework represents a shift in the conventional risk assessment paradigm, such that assessments would now: (1) focus on identifying at-risk communities as opposed to hypothetical individual risks for the reasonably maximally exposed individual from point sources or other environmental or product exposures ; (2) use qualitative/semi-qualitative data (e.g., general exposure indicators, severity rankings, non-quantitative information on known stressor interactions), and (3) incorporate non-chemical stressors. Many of these ideas were further developed, along with proposed methods for meeting cumulative risk evaluation objectives, in a 2007 US EPA report called, “*Concepts, Methods, and Data Sources for Cumulative Risk Assessment of Multiple Chemicals, Exposures, and Effects: A Resource Document*” [15]. Also, the recent NRC Science and Decisions report reemphasized the need for these shifts and reiterated many of the same cumulative risk assessment principles [6]. The report also highlighted that, to date, consideration of non-chemical stressors in the context of background stressors has been limited.

Some aspects of cumulative risk assessment have been conducted for decades under the purview of the Comprehensive Environmental Response, Compensation, and Liability Act (CERCLA) of 1980, also known as the Superfund program, which was designed to address risks from multiple pollutants across multiple pathways for residents living near hazardous waste sites [16]. This has mainly been accomplished through a site investigation to identify chemicals of concern for relevant exposure pathways and calculating non-cancer and cancer risks for specific receptors. CERCLA requires consideration of “susceptible” receptors as part of the risk assessment process, which generally includes potentially exposed children, elderly people, or people with pre-existing health conditions. This is usually accomplished by identifying the reasonable maximally exposed (RME) individual and through the application of toxicity criteria that are designed to be protective of the general population, including sensitive sub-populations [1,17]. In general, dose additivity is assumed, both for exposure pathways and chemicals, although there are provisions to segregate risk by target organ if appropriate [1]. While considering multiple on-site exposures, risk assessments conducted under CERCLA or other remediation-oriented programs do not often take into account non-point source chemical exposures (e.g., lead paint) or other community stressors like poor nutrition, obesity, or presence of psychosocial stress factors when evaluating health risks.

Although the consideration of multi-chemical, multi-pathway risks in site-specific risk assessment is routine, risk assessments that support the regulation of specific chemicals or processes (e.g., pesticides, food additives, product safety) are usually conducted in isolation, and do not include background chemical exposures or exposures to additional chemicals or chemical sources. Human studies include individuals (both in the control and chemical-exposed groups) who have background exposures; as a result, the interactions between background factors and the exposures under study are rarely evaluated in any meaningful way (*i.e.*, findings are usually focused on the chemical exposure). One of the goals of cumulative risk assessment is to address this limitation, and to expand evaluations to include background conditions (from chemical or non-chemical stressors) and how they relate to additional exposures that could contribute to increased health impacts.

More formal cumulative risk assessments at the agency level are largely focused on chemical-chemical interactions, likely because information on chemical-chemical interactions are more data-rich than information on chemical-non-chemical interactions. For example, a frequently cited example of cumulative risk assessment is the evaluation of aggregate exposures to pesticides mandated by the Food Quality Protection Act of 1996, which specifically states that pesticides with a common mechanism of action be evaluated for their cumulative health risks [18]. To meet this requirement, a cumulative risk assessment has been conducted for organophosphate (OP) pesticides, which is a class of pesticides that have a common primary mechanism of action, acetylcholinesterase (AChE) inhibition [19,20]. The OP cumulative risk assessment considered multiple OPs simultaneously across multiple exposure pathways. Based on a common mode-of-action (MOA), US EPA was able to assume dose additivity. Currently, a multi-chemical cumulative risk assessment is in development for pyrethroid pesticides (type I and type II) [21]. Unlike OPs, this group of pesticides does not have a unified MOA, and has known interactions with other pesticide classes, which has hindered progress and highlights some of the complexities involved in the evaluation of multi-chemical exposures, when additivity cannot be assumed.

Another example of US EPA’s efforts to implement multi-chemical risk assessment is through the National Scale Air Toxics Assessment (NATA) [22]. The goal of this program is to evaluate sources, levels, and potential risks of hazardous air pollutants (HAPs). By modeling emissions from a variety of different source types, including major stationary sources, area sources, and on-road and off-road mobile sources, the NATA results provide estimates of airborne exposure concentrations and associated outdoor inhalation risks for small geographical areas (*i.e.*, at the county and the census-tract level). Both cancer and non-cancer endpoints are evaluated, although non-cancer endpoints are restricted to respiratory and neurological effects. US EPA has made the conservative assumption that effects of multiple compounds on the respiratory or nervous system will be dose-additive and, thus, a hazard index for each of the non-cancer categories is calculated. To date, three comprehensive assessments have been conducted based on emission data from 1996, 1999, and 2002 [23–25]. The 2002 assessment includes emissions, ambient concentration estimates, and exposure estimates for 181 of the Clean Air Act’s 187 “air toxics” substances (plus diesel particulate matter).

These assessments, which are often cited as some of the more robust examples of cumulative risk assessments, consider only multiple chemicals and multiple routes of exposure, but do not include consideration of non-chemical stressors (inclusion of non-chemical stressors may be included in cumulative risk assessments, but are not required under US EPA’s definition of cumulative risk). Initial attempts to achieve the goals of the incorporation of non-chemical stressors are only now beginning to be developed, particularly in epidemiological research, and preliminary efforts have been successful in identifying key hazards and exposures, but integration of these components (as is usually accomplished under the traditional risk methodology) is extremely complex. Some of the complexities identified in these initial efforts, however, will eventually move the science forward.

## 3. Vulnerability in the Context of Cumulative Risk Assessment

The integration of non-chemical stressors into a cumulative risk assessment framework has been largely driven by the extensive evidence that there are large disparities in health across different segments of society [9,26–28]. Both US EPA [14] and NRC [6] have focused discussion of incorporating non-chemical stressors around the notion of vulnerability. Additionally, the environmental justice movement, which has been influential in initiating and shaping the direction of cumulative risk assessment, is premised on the concept that poorer communities are vulnerable both because they carry a disproportionate amount of the environmental burden and, by virtue of their social environments, are uniquely sensitive to environmental pollutant exposures [4].

The consideration of vulnerable or sensitive populations in human health risk assessment is not new. Conservative inputs in risk assessments are standard practice to ensure protection for the most sensitive population groups. Risk assessments and health-based policy have often used terms like “sensitivity,” “susceptibility,” and “vulnerability” interchangeably, often referring to any condition that increases the probability of an adverse health outcome. In the context of cumulative risk assessment, the attributes of vulnerability have been more clearly articulated. Recent literature has made some distinctions between the different facets of vulnerability, which both US EPA [14] and NRC [6] believe should be considered as a part of the cumulative risk assessment paradigm [26].

In the establishment of National Ambient Air Quality Standards (NAAQS), US EPA is mandated under the Clean Air Act to provide a margin of safety to protect sensitive sub-populations by considering vulnerability and susceptibility factors in its health assessments. Specifically, Section 109(b) (1) of the Clean Air Act defines a primary standard as “the attainment and maintenance of which in the judgment of the Administrator, based on [air quality] criteria and allowing an adequate margin of safety, are requisite to protect the public health.” Furthermore, the legislative history of Section 109 indicates that a primary standard is to be set at “the maximum permissible ambient air level…which will protect the health of any [sensitive] group of the population,” and that for this purpose “reference should be made to a representative sample of persons comprising the sensitive group rather than to a single person in such a group” [29]. Table 1 summarizes the vulnerability and susceptibility factors that US EPA has identified as being associated with specific criteria pollutants. In general, the NAAQS reports have distinguished between sensitivity (*i.e.*, biological factors, including age or gender) and vulnerability (*i.e.*, non-biological factors, such as SES and proximity to roads). This grouping is similar to the Kasperson scheme highlighted in US EPA’s Cumulative Risk Assessment Framework [14], as discussed below, but does not overlap completely—introducing some confusion. This confusion is magnified by the blanket use of the term “susceptibility” to describe both susceptibility and vulnerability factors [30,31]. As discussed in Section 5.2, the lack of a proper definition and the disjointed way in which these factors are assessed in air pollution studies makes it difficult to evaluate and use the results in any quantitative fashion. In fact, although this information is summarized and discussed extensively in the supporting documentation for the NAAQS reviews, it is not clear how this information is used by US EPA in the establishment the NAAQS.

Although not developed specifically for cumulative risk assessment, US EPA presented a framework developed by Kasperson for differentiating among different types of vulnerabilities: (1) susceptibility and sensitivity; (2) differential exposure; (3) differential preparedness; and (4) differential ability to recover [14]. In terms of human health cumulative risk assessment, it is useful to think of these vulnerability factors as being related to innate biology or genetics (susceptibility and sensitivity), disproportionate chemical burden (differential exposure), and social factors (differential preparedness and recovery). These vulnerabilities may either be related to the individual’s attributes or may reflect community features that bear on individual outcomes, although some vulnerabilities, such as socioeconomic status (SES), act on both the individual and community levels.

### 3.1. Susceptibility and Sensitivity (Vulnerability Related to Biological Characteristics)

Susceptibility and sensitivity describe innate biological conditions that make an individual or sub-population more likely to experience adverse effects from a chemical exposure compared to the general population. Susceptibility to environmental insults may be due to life stage (e.g., developing fetuses, children, the elderly, pregnant women), underlying diseases, and/or genetics, including polymorphisms [14].

Traditionally, risk assessments have considered these susceptible sub-populations in development of quantitative toxicity criteria, with potential differences in sensitivities among people incorporated through the application of uncertainty factors [*i.e.*, usually a 10-fold uncertainty factor (UF) for intraspecies variation]. According to US EPA [17], “The intraspecies UF is applied to account for variations in susceptibility within the human population (interhuman variability) and the possibility (given a lack of relevant data) that the database available is not representative of the exposure/dose-response relationship in the subgroups of the human population that are most sensitive to the health hazards of the chemical being assessed.” Several studies have evaluated the protectiveness of the 10-fold UF for interindividual variability [37]. Based on an evaluation of the variability in pharmacokinetics and pharmacodynamics within a population, Burin and Saunders [37] concluded that a 10-fold safety factor is protective of greater than 99% of the human population. This is consistent with conclusions reached by Dourson *et al.* [38], who concluded that, when based on studies in populations with sensitive individuals, current methodologies are protective for close to 100% of the general population.

Understanding the biological susceptibilities to disease has been an active area of research for many years, particularly in the context of early-life exposures. More recently, researchers have made advances in understanding the complex interactions between genes and the environment and are attempting to quantify how underlying existing disease is influenced by environmental exposures. These concepts can be incorporated into cumulative risk assessment once they are better developed, but at present, cumulative risk assessment efforts are more focused on addressing other facets of vulnerability, including differential exposure and coping mechanisms, as discussed below. While it is important to understand biological vulnerabilities, and adjustment of chemical toxicity factors may be warranted, these factors are inherently present within all populations and are thus not subject to risk management or regulation. Examples of vulnerabilities related to innate biological characteristics, as well as facets of vulnerability that are more central to current cumulative risk assessment efforts, are presented in Table 2.

### 3.2. Differential Exposure (Vulnerability Related to an Increased Chemical Burden)

Vulnerability from differential exposure relates to the concept that an individual or population may be disproportionately affected by a chemical because of past chemical exposures or increased contemporaneous exposure that increases the baseline body burden [14]. Differential exposure is related to the environmental justice movement’s concerns that disadvantaged communities are more likely to experience increased exposure to higher levels of environmental contamination (e.g., landfills and hazardous sites, industry emissions, vehicle emissions, *etc.*). Methods for quantifying differential exposure to multi-chemical stressors that can be used in cumulative risk assessments are being developed on multiple fronts, including the use of biomarkers and national databases to characterize chemical exposures. The advantages and limitations of these methodologies are discussed in Section 4.2.

### 3.3. Differential Preparedness and Recovery (Vulnerabilities Related to Social Environment and Behavior)

US EPA [14] described differential preparedness as the ability of an individual to withstand the insult of a chemical stressor based on existing coping systems and resources. Differential preparedness, therefore, relates to the potential vulnerabilities associated with social environments, including all aspects of psychosocial stress. Neither US EPA [14] nor NRC [6] has proposed a formal definition of psychosocial stress, although working definitions have been established by others. For example, the National Institutes of Health (NIH) stated that “[p]sychosocial stress refers to acute or chronic events of psychological or social origin which challenge the homeostatic state of biological systems” [41]. Under this broad definition, psychosocial stress can manifest itself in many forms. These may include the stress from living near a hazardous waste site or a noisy airport (also a physical stress), as well as numerous stressors often associated with low SES, such as exposure to violence, unemployment, and/or an unstable family structure [6,14,42,43]. Also, differential preparedness might relate to secondary manifestations of the social environment that increase vulnerability, such as poor nutrition, substance abuse, obesity, and/or smoking. Preventive health care access can also play an important role. While it is not feasible to present an exhaustive list of possible factors that contribute to differential preparedness to a chemical stressor, Table 2 lists examples gathered from several different publications.

Vulnerability due to differential recovery, described as the ability to recover from the effects of a stressor [14], is distinct from differential preparedness, but likely depends on many of the same social factors. Psychosocial stresses, poor nutrition, substance abuse, *etc.*, may also affect an individual’s or a community’s ability to recover from a chemical exposure. Especially important in differential recovery is health care access. Some of the key stressors associated with differential recovery are also presented in Table 2.

As noted above, these types of stresses, in particular, are often viewed as having an impact on the individual or the community, although overlap occurs (e.g., Personal SES *vs.* Community SES). This becomes an important distinction because the relationship between the social stressor and health outcome for an individual may manifest itself differently at the community level. Also, quantifying the relationship between disease and individual *vs.* community stresses, as well as the application of risk mitigation measures, will likely need to proceed along different lines of research.

## 4. Cumulative Risk Assessment and the Traditional Risk Assessment Paradigm

As stated earlier, accounting for increased vulnerability and sensitivity is not new to risk assessment, but consideration of these issues to date has been mainly accomplished through conservative exposure assumptions and the application of standard uncertainty factors to toxicity criteria, a relatively blunt, but health-protective approach that has mainly focused on age-related susceptibilities and the heterogeneity in a population’s response to a chemical exposure. Moving beyond these conventional approaches requires consideration of how non-chemical stressors fit into each stage of the present risk assessment paradigm, as proposed by the NRC in 1983: Hazard Identification, Dose-Response Assessment, Exposure Assessment, and Risk Characterization [44,45]. Currently, cumulative risk assessment efforts are community-level initiatives that use community information as a basis to build the risk profile, mainly in the identification of potential stressors and/or potentially vulnerable populations groups (see Section 4.4). In contrast, traditional risk assessments focus more on exposure sources (*i.e.*, they are source-centric).

To meet the goal of community-based assessments, it is important to draw from multiple fields that have tackled similar issues. For example, some researchers have proposed drawing from ecological risk assessment methodologies, including the use of multi-level analyses that incorporate individual- and community-level effects [39,46]. The complexity of such analyses can be daunting, requiring more explicit elucidation of risk assessment goals and coordination of information from several different scientific disciplines, including, for example, toxicology, epidemiology, nutrition, neurotoxicology, and the social sciences.

At this stage in the development of a cumulative risk framework and methodology, it is unclear if more research is needed to identify and define non-chemical stressors such that they can be incorporated into the existing risk assessment framework or whether a new cumulative risk assessment paradigm must be developed to accommodate the effects of non-chemical stressors. In the 2007 US EPA report, “*Concepts, Methods, and Data Sources for Cumulative Health Risk Assessmen*t,” US EPA scientists explored new paradigms for evaluating cumulative risks, and, while aspects of the traditional risk framework (*i.e.*, the NRC’s 1983 risk scheme) are still central to cumulative risk assessment, US EPA proposed additional steps (e.g., steps involving planning and scoping, problem formulation, and supplemental economic, political science, and social analyses). These additional components represent an attempt to expand the focus of risk assessments beyond identifying the risks associated with sources of contamination to understand all the key potential risks (real or perceived) that a community may face.

The section below discusses how cumulative risk principles fit into the traditional risk assessment paradigm, with examples of progress to date and research needs for both community-based and individual risk assessment. While this framework is not sufficient to accomplish all the goals of cumulative risk assessment, as noted in US EPA [15], it is likely that core elements of the traditional risk framework will need to be integrated throughout cumulative risk assessment efforts as it develops. Additionally, we provide a review of current research on how non-chemical stressors can modify the health effects of air pollution, as well as future research needs for application of these data for cumulative risk assessment.

### 4.1. Hazard Identification

The first stage in risk assessment is identifying environmental agents that are associated with known health effects. The scientific disciplines of epidemiology and toxicology have been central to this undertaking, with decades of research being devoted to understanding the link between chemical exposures and disease on the molecular, cellular, individual, and population level.

The health risks associated with non-chemical stressors, particularly indicators of low SES, have been studied extensively, and there is some information to suggest that the biological basis for these health effects is due to the stress associated with many psychosocial factors (*i.e.*, vulnerability related to differential preparedness and recovery). Much remains unknown, however, particularly in regards to the relative contribution from multiple non-chemical stressors to disease. Importantly, research on the interaction of chemical and non-chemical stressors on various health impacts is still in the early stages, as discussed in more detail below. This may be one reason why neither US EPA [14,15] nor NRC [6] has published a list of potential non-chemical stressors and associated health outcomes that should be considered in cumulative risk assessments. Instead, non-chemical stressors have been introduced inconsistently in peer-reviewed publications, often by example. Many of these are presented in Table 2.

Much work remains on the identification of potential non-chemical hazards and their associated health impacts. As with traditional chemical risk assessments, information from the fields of toxicology (and other biological sciences) and epidemiology (and other statistic-based sciences) will be needed to identify causal links between non-chemical stressors and disease. In addition, due to the large number of stressors (both chemical and non-chemical) that could be considered, methods for prioritizing or identifying key stressors will be needed to streamline and reduce the complexity of cumulative risk assessments. The sections below summarize some of the key non-chemical stressors that are currently being considered in the context of cumulative risk assessment efforts.

#### 4.1.1. Physical and Biological Stressors

In general, non-chemical stressors are divided into physical, biological, and social factors. Attention to physical and biological stressors in US EPA [14] and NRC [6] reports has been cursory and lacks clear definition. Physical stressors include radiation, noise, vibration, odor, temperature, and humidity [6,14]. Biological stressors largely encompass pathogenic agents (e.g., bacterial and viral agents).

Compared to social stressors (described below), incorporating physical and biological factors in a cumulative risk paradigm is likely more feasible in the near-term, mainly because of the availability of information on biological interactions among stressors, established metrics to evaluate exposure, and existing risk assessment methodologies for some biological and physical stressors. Also, many of these stressors are associated with specific health endpoints and even have a well-defined MOA, making them easier to evaluate in the existing risk assessment framework. Yet, integrating this information into a risk assessment framework has not come to fruition. For example, the scientific literature is replete with information on radiation (both ionizing and ultraviolet) and chemical interactions, which have been described at the molecular, cellular, and organism levels. Radiation (ionizing) risk assessment is particularly well-developed, and yet, radiological risks are generally kept separate from chemical risk assessment, including under the Superfund program [1,47].

Noise-chemical interactions are also well-studied, particularly in the context of occupational health. While there is likely enough information to understand the biological underpinnings of such interactions, incorporation of cumulative effects in a risk assessment framework has not been accomplished [48].

Initiatives to address the intersection of biological and chemical risk have been most robust under the risk evaluation of biosolids. While there are well-developed chemical and pathogen risk assessment methodologies, when NRC evaluated biosolid risk, it concluded that “because of data gaps and lack of risk-assessment methods for complex mixtures, it is not possible at this time to integrate pathogen risk assessment with chemical risk assessment” [49].

Pathogen-chemical risks have been evaluated in the context of the addition of appropriate disinfectants to drinking water supplies; however, these assessments have mainly been evaluated as risk trade-offs rather than cumulative impacts. In addition, information applicable to risk assessment that can be garnered from chemical immunotoxicity studies is available, particularly studies that involve pathogen challenges, but, again, implementation of this information in any formal context remains uncharted.

For other physical stressors, such as humidity and temperature, information on their relationship with specific health endpoints is available, but these effects have not been included alongside chemical exposures in a risk assessment framework. From an exposure standpoint, quantifying these types of exposures in a given population is relatively straightforward [6], but more work would be needed to quantify the effects of these physical stressors on specific disease endpoints. In addition, further research is needed on potential interactions, *i.e.*, whether these are additive (no interaction) or are capable of exhibiting some elements of synergy or antagonism.

#### 4.1.2. Social Stressors

While US EPA and NRC frameworks clearly state that both non-chemical and chemical stressors can be considered in a cumulative risk assessment, this paper will focus on identification, quantification, and characterization of social stressors specifically. This emphasis, as discussed earlier, is consistent with the focus of most cumulative risk programs currently under development [e.g., California Environmental Protection Agency’s (CalEPA) and New Jersey Department of Environmental Protection’s (NJDEP) Cumulative Impacts initiatives], where the goal is to identify populations with greater than average chemical burden (differential exposure) and that are more likely to experience adverse health effects by virtue of their social conditions (differential preparedness and recovery).

Psychosocial and related stressors are acute or chronic events of psychological or social origin that challenge the homeostatic state of biological systems. Social stressors could also include behaviors associated with psychosocial stress, such as poor diet, obesity, smoking, and/or illicit drug use. Stress from living near a pollutant source (e.g., hazardous waste site, power plant, *etc.*) has also been discussed as a psychosocial factor potentially contributing to increased vulnerability to disease.

Several researchers have found it useful to distinguish between social stressors that are primary to the individual *vs.* community-wide social stressors [13,40]. Although there is overlap, this division is valuable in the context of cumulative risk assessment, as it can help focus research needs on the factors that can be evaluated at the individual level *vs.* those that can be used to assess associations between stressors at a population level. Table 2 groups social stressors affecting the individual and the community.

Identifying the gamut of possible social stressors is only one half of the equation in the hazard identification step; the second half involves understanding the relationship between social stressors and a specific health endpoint. NRC [6] proposed two approaches for identifying stressors for inclusion in a cumulative risk assessment: effects-based and stressor-based. The effects-based approach begins with an effect of concern, such as elevated respiratory disease or other health problems of interest in a population [46]. This approach retrospectively uses epidemiological evidence or surveillance data to identify populations with increased disease, with the objective of understanding the stressors that contribute to that disease endpoint. Stressors of interest are then identified and assessed both individually and in combination. Aspects of this approach are borrowed from ecotoxicology, where, in general, multiple influences, chemical and otherwise, are considered in assessing total community impacts. For example, US EPA has developed a framework for identifying a diverse array of stressors that may impact water bodies that takes in account information on chemical (e.g., elevated concentrations of metals or ammonia), physical (e.g., increased sediment or water temperature), and/or biological (e.g., increased abundance of an invasive species) stressors [46,50].

In contrast, the stressor-based analysis is more prospective and begins with the stressors, then identifies the populations that may be affected. The concept of this approach is similar to traditional risk assessment, but in this case would go beyond point-source exposures and would include background exposures and non-chemical information. NRC [6] suggested that this approach can be used in conjunction with an assessment of different risk management options to identify a key subset of stressors of concern and their potential link to health outcomes of interest (*i.e.*, choose only those that would be affected differentially by the risk management strategies or would otherwise have an influence on risk estimates). As a simple example, assessment of risk from living near an airport where air pollution (chemical) and noise (non-chemical) both potentially affect hypertension, would focus on reduction of both air pollution and noise if an interaction between the two stressors is associated with increased risk.

Some researchers have suggested a life-course approach that incorporates the combined effects of multiple stressors across a lifetime (from gestation through childhood, up to later adult life) to address chronic health effects [51]. Although the concept may be simple, implementation is complex and limited to specific knowledge, not only of the physiologic trajectory of normal systems, but also the impacts of stressors at various life stages. This approach, however, does aid in the organization and conceptual framework of a complex cumulative assessment.

In response to the difficulty in isolating and testing the social stressors associated with health effects, several early attempts at incorporating social stressors into cumulative risk assessment have used indicators of social stress as a proxy for the actual biological stressors associated with disease. For example, low SES or poverty is not in and of itself a causal factor for a specific disease, but many of the attributes of financial instability may influence disease outcomes (e.g., access to health care, nutritional deficiencies, stress of living in a violent neighborhood). Understanding the biological mechanisms underlying the causality of social conditions-disease interaction may not be necessary for preliminary cumulative risk applications, but, ultimately, from the perspective of intervention and regulatory strategies to improve health, it will be important to have a more precise understanding of how and to what extent social “hazards” affect disease incidence. For this reason, the incorporation of social stressors into cumulative risk assessment is progressing along two fronts, *i.e.*, in assessing risks at the biological level and in assessing community-level risks.

At the community level, researchers have made efforts to identify key demographic variables that contribute to disease. Much of this research already exists, but draws from scientific disciplines that are not traditionally associated with chemical risk assessment, such as social science and psychology [52,53]. Most of this epidemiology-based research involves establishing statistical associations with specific social stresses and specific health endpoints, which is how hazards are identified. As detailed in Section 5.2, however, understanding these associations in the context of environmental exposures is complex.

Although less well-developed, there is also research aimed at understanding the biological responses of social stresses and their relationship with disease. Studying the biology of social stressors is significantly more complex than evaluating chemical, physical, or biological agents associated with adverse effects for several reasons. First, social stressors do not exist in isolation; often, different social stresses are directly correlated and it is difficult to segregate individual stresses to understand disease relationships. For example, how do we understand the relative contribution of poor nutrition, obesity, and stress from exposure to violence in an underserved population? Second, many social stressors are not easily measured in a laboratory setting. Additionally, several stressors in experimental animals do not have a clear counterpart in human populations. For example, experimental animals are routinely confined to small spaces, are often housed singly, and are often denied sex. Thus, results in experimental animals may not be representative of humans who are not stressed in these ways. While animal models have been useful in studying some aspects of social stress (e.g., malnutrition, noise), they are clearly limited for examining uniquely human conditions, such as the stress of being unemployed or having poor access to healthcare. The use of animal models to study social stress will be discussed in more detail in Section 4.3.

Importantly, most of the existing research on the association between social stresses and disease has not incorporated potential interactions with chemical exposures. Thus, even if a social stress can be identified as causal in a disease pathway and is considered a “hazard,” the modifying effects of social conditions on chemical exposure effects are only now being studied in a limited fashion and much is still unknown. In Section 5, we summarize some of the current research on the potential modifying effects of social indicators on air pollutant-related health outcomes. This research, which is critical for the advancement of cumulative risk assessment, will help in developing scientifically sound community-based assessments that incorporate biologically relevant stressors. This will entail investigation of specific social indicators, specific exposures, and specific health outcomes. Initial efforts in this area will be discussed below in the context of air pollution research.

### 4.2. Exposure Assessment

Exposure assessment is the step in the risk assessment process where the magnitude, duration, and spatial extent of exposure are defined [44]. In traditional risk assessment, which is based on understanding the incremental risk from chemical exposures, usually from a particular source, little consideration is given to existing underlying community exposures that may influence the toxic threshold of point-source exposures.

Accounting for existing and incremental exposures (and thereby assessing total body burden) and the relationship to overall risk is a key goal of cumulative risk assessment. In this context, most work in cumulative exposure assessment has focused on using geographic information system (GIS) databases and other general sources of environmental data to identify communities with disproportionate exposures to environmental contamination [54,55]. This may include sources of environmental data, such air pollutant concentrations, which may provide relatively good information about expected exposures on a community level, but mostly includes indirect indicators of potential exposure. For example, the Toxics Release Inventory (TRI) emissions data, location of hazardous waste sites, the presence of landfills, national radiation data, and Brownfield development information. Types of information that are being used as indicators of chemical “over-burden” are listed in Table 2 and Table 3.

Much of the current community-based cumulative exposure assessments focus on consolidating information on exposure indictors into a central database that can then be used to rank communities with potentially high environmental burdens. On the national level, US EPA’s Community-Focused Exposure and Research Tool (C-FERST) reflects the most comprehensive effort to integrate publically available chemical information for use in community-based cumulative risk assessments. At this time, this tool is focused on evaluating cumulative chemical exposures, although it has been recognized that non-chemical stressors (or indicators of non-chemical stress, *i.e.*, noise, SES, race, *etc.*) should eventually be incorporated into exposure models [55].

Similar work is being developed by US EPA’s Office of Enforcement and Compliance Assurance (OECA) (discussed in more detail in Section 4.4). These tools offer a chance to screen for areas with the potential for environmental contamination, but challenges remain in how to best leverage community-level exposure information to understand what factors, and in what proportion, they best predict individual risk. For example, while the presence of a landfill might be used as an indicator of the potential for chemical exposures, operational practices and pollution controls vary by facility; therefore, a large amount of uncertainty remains associated with how a specific facility might (or might not) ultimately impact a nearby community.

A promising alternative approach, which may provide a more direct measure of “exposure” to chemical and non-chemical stressors, is the use of biomonitoring. The potential usefulness of biomonitoring in a cumulative risk context has been emphasized by US EPA [15]. Traditional risk assessment has used biomonitoring either through the measurement of a single constituent in an appropriate biological media or through the measurement of disease biomarkers to understand the relationship between chemical exposure and disease [56]. Lead risk assessment is a current example in which risk determinations and risk interventions are often based on blood lead levels—a measurement of cumulative lead exposure from all sources [57].

Moving beyond a single-compound exposure is, of course, central to cumulative risk assessment. In terms of biomonitoring, measuring the combined exposure of multiple stressors may best be accomplished through the activation of a common biomarker, such as a biomarker of exposure (with no direct toxicological consequence), but preferably through the activation of a key biological response involved with the toxicological MOA. In other words, it would be ideal to identify an early (hopefully reversible) biological endpoint that becomes activated in response to a diverse set of exposures (both chemical and non-chemical).

The idea of examining biomarkers from multi-chemical exposures is not new. For decades, there have been attempts to assess total DNA damage by looking at chromosomal damage or DNA adducts in workplace environments. In many cases, evaluating a general biomarker of DNA damage was done in order to identify hazardous industries in which there was limited knowledge of the suite of chemicals causing adverse effects [58–60]. This approach has been particularly prevalent in industries with exposure to multiple polycyclic aromatic hydrocarbons [61].

The lessons learned from biomarker monitoring in the workplace are relevant to cumulative risk assessment in communities. From a hazard identification perspective, understanding how overall exposures may converge on a precursor to disease may be useful in targeting areas for future study. In terms of risk characterization, however, it can be difficult to identify the most significant exposures and design interventions when assessing cumulative exposure to multiple chemicals through a common biomarker.

This has been an issue in controlling workplace exposures, but would be even more problematic for identifying community exposures, where exposures may be diverse in time and space, and may be associated with multiple sources. Other issues with biomarkers as a measure of exposure include understanding biomarkers in the context of actual disease incidence, and the feasibility of collecting biological samples on a community-wide scale.

Much of the progress to date in cumulative risk associated with multi-chemical exposures has hinged on the idea of different exposures converging on a common biomarker. For example, the risk assessment of organophosphate (OP) pesticides represents a robust example where a common biomarker (acetylcholinesterase [AChE]) has been used to assess cumulative exposure to distinct compounds. In the context of OPs, this approach is useful because these pesticides operate *via* a common MOA and AChE induction is a key component in the disease pathway to neurological effects [19,20].

Using biomarkers to understand how social stressors may contribute to disease will be significantly more complicated, although biomarkers will likely play an important role in advancing the science. Ideally, biomarkers will offer an avenue for linking mechanistic-based research with epidemiological research.

An avenue of research that has been explored in the context of cumulative risk assessment and the response to multiple stressors is the idea of “allostatic load.” Allostasis is the process by which the body responds to environmental cues to restore homeostasis. The concept of allostatic load was first introduced by McEwen and Stellar in a 1993 publication, but the concept has been further developed since then [62]. McEwen [62] uses the term “allostatic load or overload” to refer to the wear and tear that results from either too much stress or from inefficient management of allostasis (*i.e.*, not turning off the response when it is no longer needed). Measuring allostatic load, which, in practice, is quantifying the exposure associated with stress, can be done through the measurement of a variety of different biological functions, including markers of neuroendocrine function (e.g., cortisol and epinephrine levels), immune function (e.g., Interleukin-6, tumor necrosis factor-alpha), metabolism (e.g., cholesterol, triglycerides), cardiovascular endpoints (e.g., blood pressure, heart rate), and anthropometric functions (waste-to-hip ratios, body mass index).

The best indicator of allostatic load, which can serve as a biomarker of psychosocial stress, remains to be determined. For example, extensive research exploring the relationship between measures of allostatic load and SES and cortisol has gained specific attention as a biomarker for aspects of chronic stress [63]. Seeman *et al.* [64] has reviewed the state-of-the-art information on the link between SES and allostatic load, noting that, while there is significant evidence linking a variety of biological responses with SES, much more work is needed to understand the biological underpinning of such responses, the complexity of multiple stress interaction, and the role of genetics. Moreover, the combination of indicators that best predicts interactions between chemical and psychosocial stress still requires further consideration [62].

Allostatic load may be one way to view how psychosocial stress as an exposure contributes to disease; other conditions associated with sub-optimal social environments will still require further research (*i.e.*, for effects of social elements that are not related, the psychosocial stress must be quantified in a different way). For example, the effects of poor nutrition, drug abuse, and/or access to health care are separate “exposures” that are not explicitly captured in an allostatic load model. Appropriate biomarkers that relate to social stresses, but are separate from psychosocial stress, may need to be developed on a more stressor-specific basis.

The use of biomarkers to quantify multiple forms of social and chemical stress is promising as a way to assess risks based on dose-response to multiple stressors. This is because, in a sense, exposure is being assessed through a common response. The challenge will be to converge on a response that is simply indicative of cumulative exposure or a biomarker that is early enough in the disease process to allow for sufficient intervention to reduce exposures. To our knowledge, there is no research to date that has been conducted to quantify cumulative exposures to a given class of environmental pollutants and social stresses through a common biomarker.

### 4.3. Dose-Response

The third step in traditional risk assessment is the dose-response assessment; this step is critical in quantifying the relationship between the exposure (or “dose”) of the chemical(s) of concern and the health outcome (or “response”). In chemical risk assessment, quantifying the dose-response relationship is usually the most resource- and research-intensive step in the risk assessment process, but it is a crucial step for quantifying the extent to which a chemical (or non-chemical) stressor contributes to disease. Understanding this relationship is necessary for discerning how much a decrease (or increase) in exposure impacts disease incidence. Although the National Academy of Sciences and US EPA have specifically stated that it is not necessary for cumulative risk assessments to encompass this quantitative attribute, it is difficult to imagine how the effectiveness of interventions could be assessed effectively without a metric for estimating targeted, cumulative risk reduction. Indeed, US EPA [15] includes quantifying dose-response relationship as a key step in conducting a cumulative risk assessment. Because of the complexity in quantifying dose-response relationships, the dose-response assessment is the most underdeveloped part of cumulative risk assessment.

As part of the dose-response analysis, all relevant scientific data (e.g., *in vitro* studies, animal data, and human exposure and epidemiological studies) are evaluated to characterize the shape of the dose-response, and, if possible, establish an MOA of the agent(s). An MOA analysis will outline the key steps in a disease process and help determine the possible form of the dose-response relationship. Establishing overlapping and diverging MOAs are an important aspect to cumulative risk assessment, and have been a central component of the chemical-only “cumulative risk assessments” conducted to date (e.g., OP risk assessment). As discussed below, although epidemiological evaluations are useful for establishing associations, it remains important to understand the biological underpinnings to definitively establish causal relationships between exposure and disease. When examining multiple exposures, understanding MOA is particularly important to assist in determining whether a given set of exposures acts independently or whether synergistic or antagonistic relationships exist.

The science of toxic interaction and influence on dose-response relationship has been accumulating over the last decade and has mainly been studied in the context of multiple chemical exposures. US EPA’s “*Supplementary Guidance for Conducting Health Risk Assessment of Chemical Mixtures*” [65] provides definitions for the different types of chemical interactions (e.g., additive, synergistic, antagonistic), however these definitions are overly simplistic because the definitions only describe interactions between two chemicals at specific doses. As such, use of these terms can be misleading, as they often describe an experimental outcome, rather than any intrinsic toxicological properties of the chemicals or stressors [66]. In light of this limitation, US EPA has chosen dose-addition as a “no-interaction” default for chemical mixtures, defining synergism and antagonism as more or less than what would be expected with additivity, respectively. The reason for this limited-labeling approach is related to large data gaps in our understanding of interactions; this remains a sizable challenge. Teuschler [67] presents several recommendations to advance the incorporation of toxicological data for improved chemical mixture risk assessments, including use of toxicological data on joint toxic action, statistical methods for analyzing dose-response for mixtures, and toxicological and statistical criteria for determining sufficient similarity of complex mixtures. As this science develops, in theory, these methods could be extended to the consideration of non-chemical stressors, allowing for identification of the MOA for relevant non-chemical stressors and quantification of the effects that a non-chemical stressor has on the biological response to a chemical.

A complication of defining the MOAs of non-chemical stressors (e.g., SES) remains their lack of a biological link to disease, that is, SES itself does not cause illness, but rather certain aspects associated with SES appear to contribute to disease. Often, it is not possible to isolate the single component of SES that is coupled to disease, but, as discussed in Sections 4.2 and 5, these types of relationships are being investigated, mainly through correlating non-chemical stressors with disease biomarkers, and in epidemiological studies investigating the interactions between non-chemical and chemical stressors and disease outcomes, respectively.

While quantifying chemical dose-response relationships can often be challenging, quantifying the relative contribution of chemical and non-chemical stressors will be more complex. In chemical risk assessment, characterization for dose-response relationships usually relies on a combination of animal and epidemiological data; in cases without supporting human data, it is possible to rely solely on animal data to quantify the relationship between exposure and adverse health effects. Animal-based bioassays enable the study of dose-response relationships because study design allows the researcher to control a number of confounding and modifying factors (e.g., age, gender, species, *etc.*). Also, bioassays offer the ability to assess the underlying disease mechanisms and serve an important role in establishing a causal exposure-to-disease relationship.

Unfortunately, exploring the relationships between social stresses and disease in animals is not straightforward, or even possible, because of the large number of social hazards. Certainly, it is not possible to examine a multi-faceted human stress factor, such as SES, in bioassays. However, there are several animal models that can be used to measure the biological consequences of social stress, but relating these results to uniquely human experiences remains problematic. For example, how does a rodent model examining stress created by exposing a weaker rat to a dominant rat relate to the human stress of not having a job? Nonetheless, the research that does exist on the relationship between social stresses and biological responses in rodents can be used a basis for understanding stress-chemical interactions. Presently, only a limited number of animal studies have simultaneously tested interactions between environmental chemicals and non-chemical stressors. For example, several studies have been conducted in rats to determine the combined effects of lead exposure and stress [68]. These studies are discussed in greater detail in Section 5.1. It should be noted that while only a limited number of animal experiments have specifically studied chemical and stress interactions, aspects of animal stress (e.g., lack of sexual contact, small cage size, handling) are a part of most animals studies, such that routine studies singularly focused on examining chemical effects are indirectly assessing interactions with non-chemical stress as well.

Epidemiological investigations clearly provide a richer and more easily adaptable data set for assessing quantitative relationships between social stresses and adverse health outcomes. For this reason, information about the interaction between non-chemical and chemical stressors has progressed most rapidly on this front and offers some clear benefits [7]. In fact, Levy [7] has proposed a framework for inclusion of the results from epidemiological studies in cumulative risk assessment. Furthermore, as summarized in Section 5.2, epidemiological studies have begun to consider the effects of social stressors on air pollution-related health impacts. The potential interaction between non-chemical stressors on air pollution effects estimates has been studied for various different health endpoints, including mortality [related primarily to particulate matter (PM) exposures], neurological effects (e.g., related primarily to lead exposures), asthma, and cardiovascular effects. As described in more detail in Section 5.2, the most frequently evaluated social stressor is some indicator of low SES (e.g., low income, low educational attainment, *etc.*), but other social stressors, such as exposure to violence, have also been studied [69].

There is important quantitative information that may be gleaned from existing studies that may help inform the relative importance of chemical and social factors in the disease process, particularly data from chemical evaluations applying stratified analyses or quantifying interaction terms with social stresses. Additionally, there remains a rich body of research defining the relationship between SES and health that needs be evaluated and adapted to be more compatible with traditional risk assessment. The existing research has helped to establish that interactions likely exist between non-chemical stressors and chemicals, but fewer studies have investigated the nature of this interactions (*i.e.*, the magnitude of this interaction or whether incremental risk from this diverse array of stressors may exhibit elements of additivity, synergy, or even antagonism). Also, although there are attempts to control for confounding, it is still not entirely possible to separate out all non-chemical stressors in a study relating environmental exposures to disease, and, vice versa, it is equally not feasible to separate out all environmental influences when studying the effects of a non-chemical stressor (e.g., SES) on disease. In this sense, it will be important to understand whether any deviations from additivity between a chemical and non-chemical stressor have a biological component or are an artifact of study design.

As the science moves forward, it will be important to design studies that allow for more-refined measurements of the relative contribution of chemical and non-chemical stressors to disease. Understanding relative contributions will be necessary to target the most effective public health interventions. Facets of social, environmental, and chemical exposures, however, are dynamic over an individual’s lifetime, and, thus, there is a temporal component to interactions that needs to be considered [26,40,70].

There are a number of additional challenges to incorporating non-chemical stressors in dose-response evaluations. As noted previously, susceptibility is incorporated into different aspects of the dose-response evaluation. A dose-response relationship not only quantifies the amount of chemical that is required for an effect, but it is also an expression of heterogeneity or “vulnerability” in a given population, where more-sensitive individuals respond at lower doses. In other words, as a result of our individual (biological) and population-specific heterogeneity, responses to chemical exposures exist on a gradient and form a dose-response relationship. When heterogeneity related to vulnerabilities is not adequately captured in a dose-response relationship from a single study (and particularly from animal studies), additional sensitivities can be accounted for through the use of uncertainty factors. Default assumptions of linearity or an assumption of no threshold are also meant to be protective of more susceptible population groups. The more explicit consideration of non-chemical stressors in cumulative risk assessment necessitates a re-evaluation of how heterogeneity is factored into the risk assessment and whether conventional approaches are appropriate. Care must be taken to not over-correct for vulnerability factors that are already accounted for through health-protective toxicity benchmarks.

### 4.4. Risk Characterization

Risk characterization is the stage in risk assessment where information from the hazard, exposure, and dose-response steps is combined to determine the level of risk. In traditional risk assessments, decisions about risk levels have relied on quantifying exposures and comparing them to benchmark toxicity factors. Evaluating multi-chemical exposures has mainly been accomplished by assuming chemical additivity for compounds with a common target organ (*i.e.*, hazard quotients are added to produce a hazard index and/or cancer risks are summed).

Recent efforts by US EPA have attempted to refine the methodology for cumulative chemical risk assessment by examining groups of compounds with overlapping MOAs. For example, as described earlier, US EPA’s cumulative risk assessment of OPs was based on the concept that OPs had a similar MOA (*i.e.*, all acted by inhibiting AChE, but to different degrees). This insight allowed for an understanding of how dose-response curves would change under multiple exposures, and, in conjunction with a comprehensive exposure assessment, US EPA was able to characterize the multi-pathway risks associated with multiple OPs [19,20]. US EPA has attempted a similar approach with the pyrethroid risk assessment, but, because it is unclear how the MOAs of different pyrethroids overlap, establishing combined dose-response relationships is difficult. The complexities surrounding pyrethroid risk assessment is an example of the difficulties that will be encountered in conducting cumulative risk assessments when considering many different types of chemicals and/or other stressors.

Due to this complexity, there will be a need to understand and integrate information on complex interactions among stressors through physiologically based pharmacokinetic and pharmacodynamic (PBPK/PD) research and the construction of biologically based dose-response models (BBDR). In theory, BBDR models may offer a way of synthesizing information on multiple responses, but from a practical standpoint, with current technologies, developing PBPK/PD models for a single compound is research- and time-intensive, such that multi-chemical models, let alone the incorporation of non-chemical stressors, will pose significant challenges. Some progress, however, is underway. For example, Wason *et al.* [71] developed a theoretical risk framework for evaluating cumulative risk to chemical and non-chemical stressors, using PBPK/PD models to quantify stressor impacts. They provided an example analysis with OP risk for an urban low-income population, considering the impact of pyrethroids as a chemical stressor and diet as a non-chemical stressor on OP internal dose and AChE inhibition. This work highlights the utility of computational models in cumulative risk assessment as additional PBPK/PD models are developed. As discussed in Section 4.2, there may be some opportunity to accomplish integration through the use of biomarkers, but there is still much to learn about the specific key hazards associated with social stress and the biology behind disease causality before risk characterization of this nature can move forward. There have been some rudimentary attempts to characterize the risk of social stress (in the metric of allostatic load). For example, Goldstein *et al.* [72] have developed a computer model to “predict effects of environmental and genetic alterations on allostatic load and therefore on the development of multi-system disorders and failures.” This approach is only conceptual at this point, but may offer a framework for evaluating the effect of multiple stressors (of different types) on disease progression.

Without a more complete understanding of how dose-response relationships change when integrating health outcomes from chemical and non-chemical stressors, it is difficult to move forward with the risk characterization in the traditional sense. An early attempt to examine social factors and the risks from multiple exposure sources is embodied in US EPA’s 1992 assessment of Chester, PA, which is located about 15 miles southwest of Philadelphia [73,74]. Chester has the highest poverty rate in the state and a large minority population (African American population of 65%). Additionally, Chester has a high density of waste treatment facilities (sewage, municipal, and medical waste) and industrial facilities. Chester’s high poverty rate, in conjunction with the presence of multiple potential sources of contamination, eventually led US EPA to conduct an evaluation that included a multi-route chemical risk assessment and a survey of health outcomes in the city. Although based on uncertain exposure data, the assessment showed that chemical risks were elevated (mainly from nearby facility emissions and lead exposures). Additionally, several health indictors (e.g., specific cancers, total cancer, total mortality, low birth weight) were found to be highly elevated compared to the rest of the state and the country. This first step in examining cumulative risk helped highlight important issues, but falls short of really understanding the interaction between social factors and environmental exposures in relation to health outcomes. As discussed earlier, this integration, however, has significant challenges.

More recent initiatives that seek to quantify the combined effects of chemical exposures and social stress involve relative hazard ranking methodologies (*i.e.*, identifying populations that, because of potentially high chemical exposures and social environment, may be at greater risk of disease). Understanding how a potential increase in chemical burden and the various social determinants of disease interact has been an active area of research for years, but tools for characterizing combined risks and, importantly, for targeting risk reductions, are under-developed. Presently, proposed methodologies focus on coarse indicators of social well-being and potential chemical exposures (often crudely measured by proximity to waste sites or industrial facilities) to provide a way to rank at-risk communities. Several of these ranking systems have been proposed for use by federal and state agencies to meet cumulative risk assessment goals. In general, these ranking systems aim to quantify what US EPA’s 2007 report [15] refers to as initiating factors or population descriptors. The aim is to identify communities with (1) multiple sources or releases, (2) evidence of elevated concentrations of pollutants (as measured in environmental media or as biomarkers), and (3) sub-standard health [15]. Some of the key tools that are in the process of being implemented, and their underlying bases, are discussed below.

One of the first nation-wide tools used to identify communities with potential disproportionate chemical exposure and demographics that may make them more vulnerable to adverse health outcomes was the Environmental Justice Geographic Assessment Tool. The functionality of this GIS-based tool was updated in 2010 and renamed EJView (see http://epamap14.epa.gov/ejmap/entry.html). This web-based resource, available to the general public, will generate maps of areas with information on toxic releases, water monitoring information, presence of health services, health status, and other important geographical features. Importantly, this tool simply provides requested information and does not make any attempt to combine information to gain an understanding of high-risk areas. However, in 2006, OECA unveiled a draft tool called the Environmental Justice Strategic Enforcement Assessment Tool (EJSEAT). The stated purpose of the tool is to “identify areas with potentially disproportionately high and adverse environmental and public health burdens” [75]. Even though the tool was first introduced over four years ago, the methodology has been neither finalized nor implemented in a formal decision-making process. The criteria for ranking communities is summarized in Table 3. In general, the system relies on publicly available data in four areas: environmental exposures, human health measures, compliance indicators, and socioeconomic indicators. Although a review of US EPA’s methodology is not readily available from the agency, according the National Environmental Justice Advisory Council (NEJAC) within each criterion, indicators are normalized to a score between 1 and 100, then the categories are normalized again relative to each other and averaged to achieve a raw score. Ranking is accomplished by normalizing the community-specific raw scores to each other.

The National Exposure Research Laboratory (a division of US EPA’s Office of Research and Development) has initiated the Cumulative Communities Research Program, which is also developing a tool to characterize cumulative risk, called Community-Focused Exposure and Risk Screening Tool (C-FERST) [55]. C-FERST appears to be similar in concept to EJSEAT, but is intended to provide all of the background information necessary for conducting community-based assessments. The goals of the tool will be to “assist communities with the challenge of identifying and prioritizing community environmental health issues, incorporating the latest research on the science of estimating human exposure to toxic substances in the environment” [76] and to assess “exposures and risks in a way that can be summed across chemical and nonchemical stressors in a comparable manner” [55]. According to US EPA’s website, this tool is still under development.

CalEPA and NJDEP are also in the process of developing methods to characterize community risks, although official guidance is still in draft form [2,3,5]. In principle, the approaches of both state agencies are similar (and consistent with US EPA tools), in that relatively rough indicators of both chemical and non-chemical stressors are being used to rank communities with the greatest potential for health risks. In March 2009, a report to NJDEP from the New Jersey Environmental Justice Advisory Council (NJEJAC) outlined a methodology for assessing cumulative impacts based on the approach published by Faber and Krieg, researchers who ranked communities within Massachusetts on the basis of environmental burden and social factors [3]. Later in 2009, NJDEP published an approach for moving forward with cumulative risk assessment that included an abbreviated version of the list of indicators recommended by NJEJAC [5]. The approach focused on indicators of environmental burden and did not address how to consider social elements in the ranking assessment, although NJDEP did find a strong relationship between its indicators of environmental exposure and poverty. The indicators selected by NJDEP are based on existing available data that can be mapped to relatively small exposure areas (100 square meters). These indicators are shown in Table 3.

In August 2010, CalEPA released a draft report called “*Cumulative Impacts: Building a Scientific Foundation*” [2]. As shown in Table 3, CalEPA has divided potential indicators of cumulative risk into five categories: exposure, environmental effects, public health effects, sensitive populations, and SES factors. The indictors in each category are provided by way of example and do not represent an exhaustive list of potential indicators. Consistent with other ranking approaches, CalEPA intends to combine these variables to screen (or rank) communities with the highest potential for cumulative risk impacts. Specially, the categories are grouped as being related to either “Pollution Burden” or “Population Characteristics.” Within each of the groups, the categories are given a score (each category has a specific range). Next, the total score for “Pollution Burden” is multiplied by the total score for “Sensitive Population.” Scores can range from 6 to 120, and, by this method, communities are ranked. In general, CalEPA noted that this approach will help identity communities with the potential to have the highest risk burdens so that these communities may be targeted for various risk-mitigating activities such as permitting, remediation, enforcement, and environmental monitoring. CalEPA also noted that this information can be used for risk assessment and standard-setting, although details on how this information would be used has not been fully developed.

In these approaches, only direct measures of exposure (instances with actual exposure point concentrations being measured or modeled) are chemical concentrations in air. This is not surprising, due to the existence of relatively extensive national (NATA) or regional (California Air Resource Board) databases for air contaminants and the infrastructure for mapping air contaminants to specific locations. From this perspective, understanding cumulative air impacts, at least in terms of chemical-chemical exposures, is more developed than understanding cumulative risks from chemicals in other media (e.g., water, soil). The measures of other forms of environmental contamination applicable to cumulative exposure remain unexplored and hypothetical. For example, as discussed in Section 4.2, it is not clear how the density of dry cleaners or living near a hazardous waste site relates to actual exposure and consequent risk.

An example where NATA data were considered in a cumulative risk assessment framework is the community case study conducted by Fox *et al.* [77] of neighborhoods in Philadelphia. Specifically, the researchers used publically available information on hazardous air pollutant concentrations (*i.e.*, US EPA NATA data) combined with toxicologically available information (e.g., the US EPA Cumulative Exposure Toxicity Database) to calculate a hazard index as well as a hazard ratio (based on LOAEL/NOAEL toxicity information) at the census-tract level, assuming additivity across HAPs. The researchers also compiled neighborhood age-adjusted cause-specific mortality statistics years potential life lost (YPLL) rates (total, cardiovascular, and respiratory). Fox *et al.* [77] assessed potential associations between mortality and YPLL rates and HAPs risk ratios for both White and non-White populations using both nonparametric ranking statistics as well as regression analyses. In the regression analyses, the authors also controlled for income. The authors found that compared to national averages the study neighborhoods had higher mortality rates and cumulative health risks across Whites and non-White populations. The correlations results suggested that there was a weak correlation between hazard ratios (based on the NOAELs/LOAELs) and mortality and YPLL rates, particularly in the non-White populations. These associations were significant only for total and respiratory mortality/YPLL, and not for cardiovascular endpoints. The authors caution that the results do not imply causality. While this case study represents a step forward in cumulative risk assessment, socioeconomic variables included in the study (e.g., racial composition and income) were limited and as acknowledged by the authors, they were not fully incorporated in the risk analyses, they were merely used as potential confounders in the regression analysis, and to test for effect modification in the ranking analysis.

Using a different approach, Su *et al.* [78] developed a Cumulative Environmental Hazard Inequality Index (CEHII) as a means of incorporating both chemical hazards and socioeconomic characteristics to asses cumulative environmental impacts. The authors used this index to assess impacts in Los Angeles County, CA. Cumulative exposures to three different pollutants were evaluated, including NO_2_ (as a marker of traffic-related air pollution), PM_2.5_ (as a marker of a secondary air pollutant with longer residence time), and cancer risks from diesel emissions. The social indicators used included racial-ethnic composition (% of population that is non-White) and income level (% of population with income 200% below federal poverty level). Cumulative risks from air pollutant exposures were assessed by both a population-weighted multiplicative and an additive approach. The combined effects of social disadvantage and cumulative air pollutant risk were assessed by calculating the CEHII, where this index provides a measure of the unequal distribution of the cumulative air pollution risks by census tract in order to rank, in a quantitative way, potential vulnerable communities (e.g., because of lower SES) that are also at greater health risks from multiple chemical exposures. This method, however, does not integrate the social indicators into the risk calculation in the traditional risk assessment way; therefore the question remains whether these communities are at increased risk because of interactions between SES status and cumulative environmental hazard index or whether these are independent risk factors. Therefore, while useful in identifying hazards and the relative contributions from several chemical exposures, the combined effects on actual health status in these communities remains elusive.

The approaches described above represent the first attempts to implement the basic principles of cumulative risk assessment. However, while these tools may be useful for identifying communities that may potentially be exposed to high levels of contamination, they are not sufficient to quantitatively characterize risk in those communities to combined effects of chemical and non-chemical stressors or to understand the relative contributions to risk of social stressors and chemical exposures. In fact, many of the criteria being identified through these programs are consistent with the initial key step (e.g., identification of initiating factors and population descriptors) proposed by US EPA [15] for conducting a cumulative risk assessment. In other words, the programs proposed by CalEPA and NJDEP may help prioritize the communities where risk assessments are needed, but, this can only be viewed as an initial step, with the more rigorous risk characterization step still in its infancy. If the goal is to identify vulnerable populations and quantify chemical risk, the ranking approach may offer an important first step, but if US EPA wants to expand the process to assess risks from multiple chemical to one that fully incorporates non-chemical stressors, more research, methodologies, and guidance will be needed. There is a particular need to apply dose-response concepts to the combined exposures to chemical mixtures and non-chemical stressors in a way that will make these risk ranking programs more useful.

Although all leading environmental agencies are explicit that cumulative risk assessment can be qualitative, a failure to root out the relative contribution of chemical and non-chemical stressors in the quantitative context may hinder efforts to move assessments that are inclusive of these social stressors forward. Under the traditional risk assessment methodology, if information on non-chemical stressors is only qualitatively characterized, the risk assessment process stalls at the hazard identification stage, and, because a dose-response is not established, it will not be possible to determine if interventions that limit specific environmental exposures will have an impact on public health.

### 4.5. Risk Management

Usually risk management is not part the traditional risk assessment methodology; however, the management-based risk assessment is one of the stated hallmarks of cumulative risk assessment [6]. Other than focusing on vulnerable communities, it is unclear how these rankings systems will translate into regulations that will improve public health. California’s Cumulative Impacts report provides some indication of planned activities in response to identifying cumulative hazards, including a more-refined permitting process for the release of toxic substances, focused remediation projects, and other activities aimed at reducing the environmental burden in vulnerable communities. What remains unresolved, however, is the feasibility of mitigating disease in socially disadvantaged communities, primarily through the control of environmental exposures. With new risk assessment tools, we can identify the general exposures and social conditions that may make a community more vulnerable, but without knowing how and to what extent these factors interact to cause disease, it will be difficult to design the most effective intervention.

The identification of the relative contributions of chemical and non-chemical stressors to disease in cumulative risk assessment offers the opportunity to consider public health more holistically such that interventions may not need to be restricted to reducing environmental exposures. Indeed, we may be able to use information from cumulative risk assessments to design policies that target the stressors (chemical or not) that contribute most to disease burden. For example, if research were able to understand the relative contribution of PM_10_ *vs.* density of community health centers to cardiovascular disease, it would allow for better-informed decisions about where to allocate resources. Of course, not all social stressors are amenable to intervention, so in the spirit of cumulative risk assessment, which is chartered to be management-based, it may be useful to focus on social stressors that can be controlled on some level, either through regulation or community initiatives.

An advantage of including social stressors in the list of possible stressors that can be controlled is that, because social stressors are multi-faceted, reducing social stress associated with a specific disease will likely benefit other elements in the social environment. To follow the example above, increasing the number of community health centers, which would have a larger impact on reducing cardiovascular diseases than further PM reductions, would likely lead to a reduction in other health endpoints as well.

Analysis of chemical and social environment interactions with disease will need to draw on research in toxicology and epidemiology, but also from expertise in the other fields, such as sociology and psychology. There is a great deal of existing research that, while not specifically aimed at informing cumulative risk assessment, will be helpful in shaping the paths forward. Importantly, as discussed in the next section, the role of epidemiological research in cumulative risk assessment is promising.

## 5. Non-Chemical Stressors and Air Pollution Exposures

In the preceding sections, cumulative risk assessment concepts were discussed within the traditional risk assessment framework and some of the major challenges associated with conducting cumulative risk assessments using existing approaches were identified. As discussed, a significant missing piece in these preliminary efforts has been quantification of dose-response for cumulative effects. As part of the dose-response evaluation, both animal studies and epidemiological studies are typically considered; however, limited information is currently available to help inform the combined effects of chemical and non-chemical stressors, and most of the research is not in a form that makes it amenable to quantifying dose-response interactions. Nonetheless, this research serves as an important foundation for future studies. The following sections summarize some of the available research from animal and epidemiological studies that have evaluated the modifying effects of non-chemical stressors on chemical effects. This summary is not meant to be an exhaustive review of the literature, but reflects much of the current research, which has primarily been conducted on air pollutants and on markers of lead exposure (e.g., blood lead and bone lead measures).

### 5.1. Animal Studies Examining the Cumulative Effects of Exposure to Chemical and Non-Chemical Stressors

Our literature search uncovered only a limited number of animal studies that have evaluated the interaction between chemical and non-chemical stressors. For example, several studies have been conducted on rats to determine the combined effects of lead exposure (only *via* the oral route) and stress (as reviewed by Cory-Sletcha *et al.* [68]). In these studies, the authors assessed changes to the hypothalamic–pituitary–adrenal (HPA) axis, the system that coordinates the body’s physiological response to stress, in the offspring of female rats that were exposed to lead and stress, both alone and in combination. The HPA axis effects were measured *via* corticosterone (the rat equivalent of cortisol) and neurotransmitter levels; behavioral effects were also evaluated. Two types of stress were used: restraint stress and cold stress. Significant effects were reported for stress and lead independently, and for the combined exposures. However, these results were significant only for some of the tested exposure time points; thus, effects were dependent on the developmental period of exposure, the timing of the measurement, the behavioral baseline, and gender. Because results were not consistent across study parameters, conclusions are difficult to draw from these studies; the findings, however, suggest that the combined effects of lead and stress are greater in female rats, compared to male rats. In addition, under certain study conditions, no effects of lead alone were observed, only in combination with stress, indicative of a potential potentiated effect of the combined exposure to stress and lead.

In another study of lead exposure, Schneider *et al.* [79] reported that rats raised in an impoverished environment and exposed to lead *via* drinking water had spatial learning deficits and decreased neurotrophic factor gene expression in the hippocampus. Rats raised in an enriched environment, in contrast, had little to no neurological deficits associated with lead exposure. The authors hypothesized that impoverished environments may exacerbate the neurotoxicity of lead, or, alternatively, an enriched environment may counter these effects. Similar results were reported by Guilarte *et al.* [80], where spatial learning deficits, as well as decreased levels of neurotransmitters and nerve growth factors, associated with lead exposure in rats, were reversed in animals that were reared in an enriched environment.

In a more recent study, Clougherty *et al.* [81] assessed the modifying effects of chronic social stress on the respiratory response to concentrated fine particles. A rat model of social stress, which involves introducing animals into the cage with a dominant male, was used as a stressor. The results suggested more severe lung function deficits associated with fine particle exposure in stressed animals compared to non-stressed animals. However, the authors cautioned that the study was limited by the small sample size. In addition, the authors pointed out that an important challenge in conducting animal studies of this kind is distinguishing between effects from acute stress and chronic stress, which have distinct physiological attributes in rats. For example, the acute stress associated with removing the animal from the cage to conduct the experiments may actually yield attenuated effects, or mask some of the chronic effects of stress.

Clearly, research in this area is in its infancy. While complicated, this research, as well as supporting studies that focus on the mechanistic underpinnings of responses, is important to advance our understanding of how the biology of non-chemical stressors and chemical stressors intersect and modify dose-response relationships.

### 5.2. Epidemiological Studies Examining the Cumulative Effects of Exposure to Air Pollutants and Non-Chemical Stressors

Air pollution epidemiological studies have begun to consider the effects of social stressors on air pollution-related health impacts. Table 4 presents some of the key studies that have evaluated social stressors in conjunction with air pollutants. The potential interaction between non-chemical stressors and air pollution effects has been studied for various different health endpoints, including mortality (related primarily to PM exposures), neurological effects (e.g., related primarily to lead exposures), asthma, and cardiovascular effects. The most frequently evaluated social stressor is a measure of low SES (e.g., low income, low educational attainment, *etc.*), but other social stressors, such as exposure to violence, have also been studied [69]. There is currently no consensus on the best indicator of social stress. Different studies use different indicators, and most often these are measures of education, occupation, and income, or some combination of these factors. Although related, they represent different dimensions of SES [82]. In addition, SES indicators are often measured at different geographic resolutions (*i.e.*, at the individual, community, or city/county level). This may explain some of the inconsistent findings across studies that have evaluated the effects of SES on air pollution health impacts, as discussed below.

Indictors of low SES have traditionally been treated as confounders in epidemiological investigations [83–85]). The definition of a confounder is a variable that is associated with both the exposure and the outcome, but is not on the exposure-disease causal pathway. Therefore, in a sense, factors such SES have been accounted for, but often not as a causal disease agent. However, researchers have begun to explore whether aspects of SES are actually effect modifiers of air pollution-related health effects [86,87]. An effect modifier is a factor that results in a change in the magnitude of an association between an exposure and an outcome when data are stratified by that factor [88]. By stratifying the analysis by non-chemical stressors, researchers can gain a better understanding of their influence and the magnitude of the modifying effect.

There is no clear consensus on how to treat social stressors in epidemiological studies, whether as confounders or effect modifiers, and the answer may be that it depends on the stressor and how it is defined and measured. Care must be taken in epidemiological studies to test stressors, particularly for confounding, as biased estimates will result if confounding is not properly accounted for. This is one of the most challenging and complicating aspects of current epidemiological efforts to incorporate non-chemical stressors. These challenges have been explored most extensively with regard to the combined effects of social environment and neurological deficits associated with lead exposure and the association between mortality and PM exposure, as discussed below.

#### 5.2.1. Mortality

A significant disparity in mortality rates exists among populations of different SES, both for all-cause mortality and for specific causes of death, such as cardiovascular disease and cancer [118,119]. Moreover, several researchers have suggested that air pollution contributes to the observed disparities in specific health effects (e.g., asthma or cardiovascular disease), leading to premature death. The two suggested hypotheses relate to the vulnerability factors discussed earlier, namely: (1) differential exposures (*i.e.*, low SES populations are differentially exposed to air pollution); and (2) differential preparedness/recovery or coping (*i.e.*, low SES populations are more vulnerable to the effects of air pollution due to, for example, poor health, psychosocial stress, or nutritional status). Most of the existing air pollution research has focused on understanding vulnerability related to the biological susceptibilities associated air pollution (e.g., pre-existing disease, age, and sex) [120–122], but, in support of environmental justice concerns, research has shifted to also consider social condition vulnerabilities [26].

A large majority of studies that have evaluated the potential modifying effects of non-chemical stressors have focused on mortality outcomes in relation to short-term and long-term exposures to air pollutants. The results from these studies, which all evaluated different indicators of SES (typically educational attainment or income), have been inconsistent, making it difficult to draw any conclusions (Table 4).

##### 5.2.1.1. Short-term Studies

Short-term mortality studies have not found consistent effect modification when analyses included stratification by SES. For example, no or weak modifying effects were observed in several large US-based studies that examined the modifying effects of SES indicators on PM_10_ mortality [87,91,92]. Similarly, in a study of residents of Mexico City (>65 years of age), O’Neill *et al.* [93] reported that ozone-related (O_3_) mortality risks did not show any consistent trends of effect modification when stratified by SES indicators. In addition, two studies conducted in São Paulo, Brazil reported contradictory results. Martins *et al.* [94] reported that respiratory mortality for PM_10_ was negatively correlated with percent college education and percent family income (>$3,500), and also reported a non-significant positive correlation with percentage of people living in slums.

In contrast, Gouveia and Fletcher [95] found greater PM_10_-associated mortality risks in districts with higher SES for residents in São Paulo, Brazil, although the results were not statistically significant. Jerrett *et al.* [89] found slightly higher relative risks across lag periods, both for mortality risks associated with coefficients of haze (CoH, a PM indicator) and for sulfur dioxide (SO_2_) in lower SES areas of Hamilton, Canada compared to higher SES areas.However, relative risks were not greatly elevated compared to the overall regional estimates (e.g., regional CoH multi-lag mortality RR = 1.06, whereas low SES RR = 1.08). Villeneuve *et al.* [96] also reported an increased percent in mortality associated with NO_2_ in a study in Vancouver, Canada for low- and middle-income families (overall percentage increase = 3.5% with 1 day lag per 17.5 parts per billion increase in NO_2_, increased to about 10%). Total suspended solid-related (TSP) mortality also increased with stratification by income levels, but similar increases were observed across all income strata. The authors stated that results should be interpreted with caution due to the small number of deaths in the income strata. No mortality effects were reported for other criteria pollutants.

In a PM_10_ study of residents in 20 US cities, Zeka *et al.* [97] found a slightly elevated percent increase risk of mortality for non-trauma mortality (0.62%, 95% CI 0.29–0.95) in a cohort of less-educated residents (<8 years of schooling) compared to more educated residents (>12 years schooling, 0.27%, 95% −0.004–0.54), although the trend was not significant. Similar results were observed for cardiac disease mortality, but not other causes of death (e.g., respiratory, stroke, *etc.*). In a study in Rome, Italy that used both income and an SES index (which included census data on education, occupation, unemployment rates, family size, crowding, and residence ownership), the authors reported higher PM_10_-related all-cause mortality for lower income and lower SES communities (1.9% and 1.4% per 10 μg/m^3^ increase in PM_10_, respectively) compared to an overall mortality increase of 1.1% per 10 μg/m^3^ for all residents [97]. Lastly, in a more recent study, Franklin *et al.* [90] assessed the modifying effects of various community-level socioeconomic variables (median household income, percent of population below poverty line, percent of adult population having graduated high school) on the mortality risks associated with PM_2.5_. Of these variables, only household income had a significant effect on the mortality estimates (specific results not reported by the authors). Similarly, for ozone-related mortality, a modifying effect was reported for community-level unemployment rates (with higher risks associated with higher unemployment) in 98 US urban communities, but not for other SES indicators, such as education, income, and poverty [98].

Given the large variability in air pollutants evaluated, together with the diverse SES variables included in the short-term studies, it is difficult to draw conclusions from these studies. Furthermore, results do not support a definitive modifying effect of SES on mortality related to air pollutants, underscoring the need for more research in this area.

##### 5.2.1.2. Long-term Studies

Two seminal US studies [the Harvard Six Cities study and the American Cancer Society (ACS) study] have found consistently elevated mortality associations with long-term PM exposures. These studies, and in particular the ACS cohort, have been the subject of extensive analysis and follow-up and have had a large influence on formulating regulation for PM_2.5_ since the PM_2.5_ standards were establishment in 1997 [31,84,123–126]. The most recent re-analysis and follow-up studies of the ACS cohort have also provided some important insight into the question of the influence of SES and other factors on air pollutant related mortality estimates. In 2000, results were published on the re-analysis of Harvard Six Cities Study and the ACS of particulate air pollution associations with mortality to address potential biases in risk results as well as the robustness of the results to model specification [84]. As part of extensive sensitivity analysis of the ACS cohort, the authors tested confounding and effect modification for a number of sociodemographic and environmental variables including several SES factors (e.g., education, income, poverty and unemployment). The results from these analyses indicated that there was no confounding effect of these ecological factors [84]. As the analysis relied on multi-level data [individual-level and metropolitan statistical area (MSA)-level covariates] in a two-stage random effects Cox model, the authors speculated that the extensive number of individual-level variables included in the first stage may have removed possible confounding effects before the ecologic covariates were tested in the second stage.

In addition, as part of the sensitivity analysis, Krewski *et al.* [84] identified potentially “susceptible” subgroups and conducted analyses stratifying by potential modifying factors. The only modifying factor that was found to have a significant effect was education, which was chosen as a surrogate of SES. In the ACS cohort, Krewski *et al.* [84] found that cardiovascular mortality was significantly associated with both PM_2.5_ and sulfates among the least-educated. For all-cause mortality, the RRs were 1.35 (95% CI: 1.17–1.56) and 1.27 (95% CI: 1.13–1.42) in the <high school education groups for PM_2.5_ and sulfates, respectively. For cardiovascular mortality, the effects in the <high school educated were 1.47 (95% CI: 1.21–1.78) and 1.39 (1.20–1.62) for PM_2.5_ and sulfates, respectively. These effects were larger than the effects reported for the complete cohorts. The difference in the relative risks may be indicative of the additional risks associated with some component of SES.

Conflicting results were reported in the recent extended analysis of the ACS cohort [85]. This analysis extends the follow-up time to 18 years (1982–2000). As in the previous analyses, the current evaluation featured sensitivity analyses that address potential confounding effects of ecologic variables (such as education attainment, housing characteristics, and level of income) on the air pollution–mortality association, but these variables were examined at both the Zip Code area (ZCA) scale, the MSA scale, and by the difference between each ZCA value and the MSA value, whereas in the previous analyses only the MSA level was evaluated. The results from this follow-up showed increased mortality risks with the inclusion of SES indicators in the model. For example, the strongest associations with all-cause mortality was reported with inclusion of the household income variable, with mortality hazard ratios of 1.048 (95% CI: 1.030–1.068) compared to the unadjusted ratio of 1.034 (95% CI: 1.1016–1.053). In the previous analysis, income had no effect on mortality risk estimates [84]. The source of the discrepancy between the results from the previous analysis and the follow-up analysis is unclear. Although in this recent analysis the authors used a finer unit of aggregation (ZCA *vs.* MSA), they also found that when they compared models that utilized different geographic units of aggregation (ZCA, MSA, *etc.*) there was no appreciable difference in the hazard ratio estimates [85]. The follow-up also assessed effect modification by education finding that for this follow-up cohort, a trend of effect modification by education was more difficult to discern and that for some health outcomes (e.g., ischemic heart disease), there was a reverse trend such that greater risks were observed for the more educated. As these results suggest, there is a need to more clearly define the role that indicators of SES play in confounding or modifying associations between health impacts and air pollution exposures.

The modifying effects of education were also examined in a French study [99]. In this study, the authors looked at all-cause mortality associated with long-term exposures to TSP, black smoke (BS), and NO_2_, finding no significant trends in mortality effects as a function of education. Similarly, education did not appear to modify the relationship between mortality and BS in a Dutch study by Hoek *et al*. [100].

Two long-term Canadian studies also evaluated the modifying effects of SES indicators on long-term air pollution exposures. Finkelstein *et al.* [101] reported statistically significant mortality associated with TSP exposures in both low- and high-income groups, with larger effects in the low-income group (RR = 1.14, 95% CI: 1.07–1.20 *vs.* 1.04, 95% CI: 1.10–1.06). Mortality associated with SO_2_ exposures were significant only in the low-income group (1.18, 95% CI: 1.11–1.26). In the second study, the authors investigated the modifying effects of a deprivation index on cardiovascular mortality related to both TSP and SO_2_ (assessed as an air pollution index), finding no significant interactions [102].

Laurent *et al.* [127] reviewed these and other epidemiological studies of the interaction between SES and air pollution-related mortality. The authors were not able to make formal comparisons between studies due to the large variety of SES indicators used across the studies. One important finding was that no effect modification by SES was found in studies that used SES indicators at coarse geographic resolutions (city or county level), whereas mixed results were reported for studies that used SES measures at finer geographic resolutions; most studies (5 out of 6) that had individual-level SES measurements found evidence of greater mortality risks in disadvantaged individuals. The authors stated that there is not enough information to conclude that SES modifies the relationship between air pollution and mortality outcomes.

##### 5.2.1.3. Cardiovascular Effects

Fewer studies have examined the interactions between social stressors and air pollution-related cardiovascular effects (other than mortality). In one cross-sectional study of 513 people with hypertension and 237 without hypertension, Peters *et al.* [109] evaluated the how stress modified the effects of lead exposure (measured by bone lead levels) on hypertension. Stress was measured using the Health and Social Behavior questionnaire, as well as measures of self-reported stress. The authors reported that the effects of lead on hypertension were more pronounced in highly stressed individuals. Results were robust to inclusion of several confounders including age, body mass index, family history of heart disease, education, smoking, alcohol consumption, physical activity, and nutritional factors.

In another study, Cakmak *et al.* [110] analyzed the interaction of SES factors with gaseous air pollutant-related cardiac hospital admissions in 10 large Canadian cities using time-series analyses adjusted for day of the week, temperature, barometric pressure, and relative humidity. The authors found that exposure to O_3_, CO, and NO_2_ were individually statistically significantly correlated with cardiac hospital admissions, with even larger combined effects. The air pollution-related cardiac effects, however, were not modified by consideration of gender or community-level indicators of SES (namely education and income). The authors concluded that the community-level indicators of SES used in the study did not identify potential susceptibility.

Several studies have looked at the correlation between cardiovascular risk factors (obesity, hypertension, smoking, and physical inactivity) and individual (educational attainment) and community-level (unemployment rate and overcrowding) SES indicators. A study conducted in Germany and the Czech Republic [128] reported that smoking was significantly correlated with areas with the highest unemployment rates in both countries, even with adjustment by individual SES factors. In Germany, obesity and low physical activity were also statistically significantly associated with community-level SES indicators after adjustment for individual SES factors. Interestingly, these effects are similar in magnitude to the cardiovascular health effects observed in air pollution studies (see, for example, US EPA [31]).

As with the mortality studies, epidemiological evidence of an interaction between air pollutant exposures, social stressors, and health is limited and inconsistent. Therefore, more research is needed to help clarify whether interactions exist, as well as the type of interaction and the potential magnitude.

##### 5.2.1.4. Neurological Effects

Controlling for confounding factors in epidemiological studies is complex and requires an understanding of all important cofactors that that can distort the true relationship between a chemical exposure and a given outcome. If the cofactor is a truly independent predictor of outcome, it can be adjusted for using standard statistical techniques. In some cases, however, the chemical and some cofactor may be so highly correlated that it is difficult to disentangle it using these standard statistical methodologies.

The complexities of the relationships between chemical exposures and the social environment have been studied extensively in the epidemiological research related to lead exposures and neurodevelopment. Several studies that have reported declines in test scores per unit increase in lead biomarkers have also observed a large reduction in these neurological impacts when adjusting for indicators of the social environment. For example, in early studies of lead effects on IQ, no effects were observed when social factors were accounted for in regression analyses [129,130]. Also, Tong and Lu [131] reported that adjustment for quality of home environment, SES, maternal intelligence, and parental smoking reduced the association between lead and intelligence quotient (IQ) by up to 40%. Similarly, in a pooled analysis of seven prospective studies, the association between lead and childhood IQ was reduced from −4.66 (95% CI: −5.76 to −3.60) to −2.70 (95% CI: −3.74 to −1.66), when variables for study site, quality of home environment, birth weight, maternal IQ, and maternal education were included in the model [132]. In fact, some researchers have determined that blood lead may account for only 1–4% of the variability in child IQ scores, compared to about 40% or more for social and parental factors [133]. More recently, researchers have questioned the adjustment of lead effect estimates by SES factors as overly conservative and have suggested instead that SES indicators are more likely to modify the association between lead and cognitive deficits [105,134]. For example, researchers point to the health effects associated with elevated glucocorticoids (a marker of chronic stress), which are also elevated with lead exposures and can affect behavioral processes. Thus, an important question is whether there is an interaction between risk factors associated with lead exposure and those associated with environmental stress, and whether these effects are synergistic. There is currently only suggestive evidence of an interaction from both animal and human epidemiological studies. In addition, SES can contribute to higher exposures of lead due to living conditions (*i.e.*, older housing) and inadequate healthcare, thus contributing to additional vulnerabilities from differential exposure and differential preparedness.

Cognitive deficits in children (measured using the Kaufman Assessment Battery) were reported to be associated with neonatal blood lead concentrations in a Cincinnati cohort of four-year-olds, but only for children from poorer families [35,103]. In a follow-up on the Cincinnati study Ris *et al.* [104] reported that SES was not a significant modifier of the higher blood lead (taken at age 78 months) association with lower learning/IQ scores, but they observed a trend of greater vulnerability in lower SES adolescents exposed to higher lead levels. This trend is supported by a study in an Australian cohort of children, in which cognitive deficits were reported to be more prevalent in lower SES groups [105]. Tong *et al.* [105] studied 375 children in South Australia prospectively from birth until 11–13 years old. The researchers evaluated the interaction between blood lead levels and sociodemographic factors [gender, parents’ occupational prestige (measure of SES), quality of the home, and maternal IQ] on children’s IQ. The authors reported statistically significant interaction with gender (*i.e.*, girls were more sensitive) and with SES measure, but these effects were reduced and became non-significant when adjusted for other covariates (e.g., age at testing, grade in school, iron status, birth weight, feeding method as infant, marital status of parents, *etc.*).

Three cross-sectional studies in Europe among preschool and school-age children found that increased lead exposure resulted in decreased IQ and decreased performance in visual-motor integration and choice reaction tests, but only in children of low SES [106]. In a similar study conducted in the US, the correlation between the Mental Development Index (MDI) and cord-blood lead levels were evaluated for infants at ages 6, 12, 18, and 24 months of age [107] stratified by social class. Social class was measured based on Hollingshead’s Four-Factor Index of Social Class, which includes a measure of parents’ occupational and educational achievements. The results showed that, at ages 6 and 12 months, there was no significant difference of effect of cord-blood lead on MDI across social status, but, at ages 18 and 24 months, differences in the relationship between blood lead levels and MDI were significant, but only at the low and medium lead exposure levels. In the infants in the high blood exposure level category, however, the differences in MDI performance were indistinguishable between social status category.

Bellinger *et al.* [107] also found that results varied depending on the age at which child was exposed to lead. For example, when blood lead levels were taken at 6 months of age, declines in MDI score with increased blood lead concentration were observed only in the lower SES group. No trends were observed when analyses were conducted using blood lead levels taken at 12, 18, and 24 months of age. The authors thus theorized that vulnerability to lead toxicity is dependent on both the infants’ SES and age. It is worth noting that the authors found that the interaction between lead exposure and SES did not show evidence of additivity or multiplicativity in these older age groups. In other words, children with the worst values on both factors (*i.e.*, highest lead exposure and lowest social stratum) did not display the worst performance, as may be expected with a synergistic effect. For example, in this study, the children with the highest cord-blood lead levels had similar MDI scores regardless of social status. The authors also stated that interactions between risk factors should be interpreted with caution due to sample size considerations. These studies highlight much of the uncertainty and limitations associated with relying solely on epidemiological studies for use in cumulative risk assessments.

Although most studies have focused on the effects of lead exposures in children, one recent study in adults found that psychosocial stress modifies the effects of lead on cognitive function in adults. Glass *et al.* [108] assessed how neighborhood psychosocial hazards, measured independently from subjects using neighborhood information on violent crimes, 911 calls, *etc.,* as a measure of a “heightened state of vigilance, alarm and threat,” may modify the effects of lead (bone-lead measurements) on cognitive function in adults aged 50–70 years old. The study, which was conducted in Baltimore, Maryland, showed that psychosocial stress exacerbated the effects of lead on three out of seven cognitive measures after adjusting for potential confounders (age, sex, race, education, technician, time of day)—namely, language, processing speed, and executive functioning.

Overall, both confounding by SES and effect modification have been reported in epidemiological studies of lead exposure and neurological deficits, primarily in studies of children, and more recently in adults. These studies provide some of the strongest evidence of potential effect modification by SES factors, but inconsistencies have been reported that underscores the need for further research.

##### 5.2.1.5. Asthma and Other Respiratory Health Effects

A growing area of research is in understanding the large disparities in asthma morbidity. The excess asthma morbidity and mortality observed in inner-city, lower-income, and ethnic minority communities in not well-understood, and the relative importance of the urban environment, lower SES, or ethnicity as independent risk factors remains controversial [27]. Current research on the modifying effects of non-chemical stressors on air pollution-related effects indicate that there is a potential interaction. For example, Clougherty *et al.* [69] assessed the potential modifying effects of exposure to violence (ETV) as a measure of a chronic social stressor on traffic-related asthma etiology. The authors used novel GIS methods to retrospectively estimate traffic-related air pollution exposures (using NO_2_ as a surrogate) for 413 children in a pregnancy cohort. Air pollution estimates were analyzed in conjunction with questionnaire-based data on ETV to assess development of asthma in the cohort. The authors found no independent effect of ETV on asthma (OR = 0.98, 95% CI: 0.78–1.22), but an elevated risk of asthma was found with increased NO_2_ exposures only in children that had higher ETV, indicating greater air pollution susceptibility. For example, in the cohort of lifetime residents, the effects of NO_2_ on asthma were positive and almost significant (odds ratio (OR) = 1.28, 95% CI: 0.97–1.69), but in the stratified cohort, NO_2_ was significantly associated with asthma among children with above-median ETV (OR = 2.33, 95% CI: 1.47–3.71), but not the children with below-median ETV (OR = 0.87, 95% CI: 0.59–1.28). Similar results were obtained when the analyses included potential confounders (e.g., maternal asthma, exposure to tobacco smoke, education, sex, and age). The authors noted the difficulties in interpreting the interactions between air pollution exposures and non-chemical stressors, such as ETV, because behaviors among people living in violent neighborhoods may differ from those living in less violent areas, such as keeping children indoors, where they may be exposed to greater NO_2_ levels from indoor sources (e.g., smoking and gas stoves) or to other indoor pollutants (e.g., indoor allergens). In addition, ETV may be a proxy for other social stressors, such as family instability.

In another study of asthma, Shankardass *et al.* [111] evaluated effect modification by low SES (using parental education) or high parental stress (measured by way of a questionnaire) on traffic-related asthma etiology. Approximately 2,500 children (ages 5–9) with no history of asthma or wheezing were followed for three years. Exposure to traffic-related air pollution was determined using dispersion modeling. High parental stress was associated with higher incidence of traffic-related risk of developing asthma. An increased risk of asthma was also observed for low SES families exposed to air pollution.

In contrast to the findings in the studies described above, in which the interaction of the non-chemical stressor with the chemical stressor appear to act in combination to increase asthma, a study that evaluated the modifying effects of life stress (assessed *via* interviews) on NO_2_-related inflammatory markers and asthma symptoms, reported greater inflammatory markers associated with greater stress at lower air pollution exposures, not higher air pollution [116]. The authors theorized that their findings may be related to a threshold effect in which the chronic stressor (in this case stress) lowers the threshold such that adverse effects occur at lower pollutant exposures. The authors also suggest that the differences observed in this study compared to previous studies may be related to the focus on children with existing asthma *vs.* the onset of asthma, and that different social stressors may have a differential effect on asthma exacerbations.

In a study conducted in 10 large Canadian cities, living in communities in which individuals have lower household education and income levels was associated with greater hospitalization for respiratory health effects, indicating that these individuals may have increased vulnerability to air pollution [112]. Stratification by education yielded significant increases in hospitalizations for the lowest educational attainment strata (<Grade 9) for O_3_ and SO_2_, but not NO_2_; the percent increase was not significantly different from the unstratified risk values. For example, for O_3_, the unstratified risk was 3.8% (95% CI: 1.9–5.6) and for <Grade 9, the risk was 4.0% (95% CI: 1.6–6.5). Similarly, the combined effects of all three gaseous pollutants yielded the greatest percent change in hospital admissions in both the unstratified and the stratified models (6.6%, 95% CI: 3.5–9.7 and 7.0%, 95% CI: 2.5–11.5, respectively). Significant risk of hospitalization stratified by income were found for O_3_ and NO_2_. The risks for NO_2_ for the lowest income level (<$21,309) were higher than the unstratified risks (5.1%, 95% CI: 1.6–8.8 *vs.* 2.5%, 95% CI: 0.2–4.8), as was the multi-pollutant effect (8.6%, 95% CI: 4.3–12.9). In a similar study of asthma hospitalizations in children (ages 6–12) in Vancouver, Canada, greater risks from asthma hospitalizations in male children of low SES were observed with exposures to NO_2_, compared to male children in the higher SES group (OR = 1.13, 95% CI: 1.04–1.23 *vs.* RR = 1.04, 95% CI: 0.95–1.14 at lag = 1 day); in female children, significant asthma hospitalizations were found for SO_2_ only at lags = 4, 5, and 6 days [113].

Disproportionate asthma hospitalizations were also observed in a study in Phoenix, Arizona using insurance status as an indicator of SES. Grineski *et al.* [114] reported that children without insurance had a 1.4 times higher risk of asthma hospital admissions, compared to those with private insurance with a 0.02 parts per million (ppm) increase in NO_2_ above the seasonal mean. Insurance status was also found to significantly modify the effect of ozone and PM_10_-related respiratory hospital admissions in New York City. After adjusting for insurance status, significant relative risks of hospitalization were reported in the uninsured subgroups, but not in the insured subgroups. In fact, these results also showed that the air pollution-hospital admission association was largely driven by the uninsured population.

The modifying effects of SES on air pollution-related respiratory morbidity are suggestive of an effects at this point, but questions remain as to the nature of the modifying effect, *i.e.*, whether these effects are additive, synergistic, *etc.* In addition, because, as with other epidemiological studies, these studies used varying indicators of SES or social stress (e.g., education, exposure to violence, and insurance status), it is difficult to compare results across studies.

##### 5.2.1.6. Limitations Associated with the Use of Epidemiological Data

The epidemiological research to date has contributed to a better understanding of potential interactions between social stressors and air pollution exposures, but more research is needed to confirm the results and resolve some of the inconsistencies across studies. Limitations and issues associated with using epidemiological results include biased effect estimates, exposure errors, and assuming causality when biological mechanisms of low-dose exposure effects are not well understood. Resolving these issues will be an important step in the current risk ranking efforts so that the best indicators of social conditions are being used to identify the communities at risk. In the absence of this analysis, cumulative risk ranking programs may be incorrectly targeting communities for further analysis. After clearer associations are established for specific indicators of social stress with specific chemicals and for specific endpoints, it will be possible to develop more specific risk mitigation measures.

As mentioned previously, epidemiological studies examining gross measures of disease are not likely, in and of themselves, to provide refined estimates of the relative contribution of chemical and non-chemical stressors to disease. It is therefore important to consider multiple lines of evidence (animal, cellular, and molecular studies) to determine which data will be most informative in elucidating MOA, as well as dose-response information. Research is moving towards trying to better understand the interactions between environmental exposures and non-chemical stressors. Similarly, as mentioned previously, studies of allostatic load (including methods for measuring allostatic load) and the effects of multiple stressors offer some promise for identifying potential MOAs [62,135]. Lastly, “molecular epidemiology” methods, which incorporate biological events at the physiologic, cellular, and molecular levels, and thus enhance the biological understanding of epidemiological findings, may prove to be useful in cumulative risk assessments.

## 6. Research Needs and Conclusions

The application of cumulative risk assessment to include the incorporation of non-chemical stressors to address environmental justice concerns requires improved or new methodologies that can be applied at the risk characterization stage of the risk assessment. Examples of robust cumulative risk assessments are available, but generally do not include assessment of non-chemical stressors in any quantitative way. In addition, a majority of environmental applications of cumulative assessments have focused on evaluating health effects associated with air pollutants, with or without consideration of population level indicators for other stressors (e.g., census level demographics, SES, *etc.*), primarily due to the relative availability of geographically based air pollutant data and basic health surveillance data (e.g., population level mortality or cancer data). Other metrics of exposure *via* other media are also being explored. In these studies, risk-ranking methods, correlating high incidence of disease with potential chemical exposures (measured often crudely by proximity to pollution sources) are typical in these assessments and are descriptive in nature. These descriptive applications serve communities by providing indicators of exposures and in identifying risk distributions across communities, and, in doing so, help to identify environmental health disparities and potentially vulnerable population groups (e.g., with the reliance on GIS methods). In this respect, these qualitative assessments remain important. Epidemiological research has progressed in the evaluation of the modifying effects of social stressors on chemical exposure-related health impacts, albeit with mixed results. This research however, offers not only a way to better understand potential interactions, but to potential quantify their effects. As with more routine single-chemical risk assessments, there is a substantial challenge in linking dose-response information gleaned from animal and *in vitro* studies with epidemiological observations. These challenges are more pronounced in cumulative risk assessment, where both the underlying biology and exposure assessment are more complex, particularly when non-chemical stressors are involved.

Challenges remain in the effort to include non-chemical stressors in the cumulative risk assessment framework in order to obtain a useful environmental and public health analysis and evaluation tool. The present inability to fully quantify risks using comparative metric(s) capable of accounting for non-chemical stressors makes it difficult to assess cumulative impacts consistently across different populations, locations, or time periods. This inability to compare risks quantitatively, which is necessary for designing and evaluating environmental health intervention programs or for assessing the effectiveness of environmental regulation, continues to impair the application of cumulative risk assessment inclusive of non-chemical stressors. In addition, to communicate intervention strategies and regulatory initiatives to any affected communities, fair and clear interpretation of risks and competing uncertainties is necessary. To advance the incorporation of non-chemical stressors into risk assessments in a manner that will facilitate effective public health interventions, research in the following key areas is needed:

- Identification of the elements of low SES that have the most significant impact on disease (e.g., to what relative extent does poor nutrition *vs.* psychosocial stress *vs.* lack of quality healthcare play a role in disease), investigated on a disease-specific basis.
- Metrics to describe degrees of psychosocial stress and other key biological effects of non-chemical stressors, specifically expressing non-chemicals stressors in manner where “dose”-response relationships can be explored.
- Correlations between gross measures of exposure (e.g., the presence of a landfill) and actual chemical exposure in a population.
- Biomarkers that are reliable indicators of the cumulative effects of chemical and non-chemical stresses.
- Correlations between stress induced in animals and psychosocial stress in humans, specifically whether these animal stress models are applicable to human conditions.
- Quantification of the interactions between non-chemical stressors and chemicals and their relative role in health outcomes. Specifically, how dose-response curves change with combined exposures to chemical and non-chemical stressors.
- Epidemiological evaluations specifically designed to explore the relative contribution of chemicals and non-chemical stressors in disease outcomes, and, specifically, how observations relate to dose-response relationships.
- Focused efforts to better “link” research on dose-response relationships to observations gleaned from epidemiological evaluations.

While much new research is necessary, it should be emphasized that there is a wealth of information to draw on from disciplines not usually associated with chemical risk assessment.

## Figures and Tables

**Table 1.** Susceptibility and vulnerability factors considered for evaluation of the criteria air pollutants.

**Criteria air pollutant**	**Susceptibility factors**	**Other vulnerability factors**	**Reference**
**Carbon monoxide**	Pre-existing disease Age Gender	Differential exposure/dose (e.g., altitude, exercise, proximity to roads) Abuse of medication and other substances SES (e.g., education and income)	[32]
**Particulate matter (PM_10_and PM_2.5_)**	Pre-existing disease Age Gender Genetic factors Race	SES (e.g., education, unemployment, and income)	[31]
**Ozone**	Pre-existing disease Age Gender Race Genetic factors	Differential exposure (e.g., activity level, time spent outdoors; physical activity) SES/racial ethnic factors (e.g., education and income) Environmental factors (urban *vs.* rural, ETV, endotoxin exposure)	[33]
**Lead**	Age Physiological states (menopause, pregnancy, lactation) Genetic factors Gender	SES (e.g., education, life stress, and income)	[34]
**Sulfur dioxide**	Genetic factors Age	SES (e.g., education and income) Differential exposure (e.g., activity level, residential location, AC use, time spent outdoors)	[35]
**Nitrogen dioxide**	Pre-existing disease Age Gender Genetic factors	SES (e.g., education and income) Differential exposure (e.g., proximity to roads)	[36]

**Table 2.** Vulnerabilities related to biological sensitivity, differential exposure, and differential preparedness and recovery.

	**Selected potential indicators of vulnerability (individual)**	**Selected potential indicators of vulnerability (community)**
**Susceptibility and sensitivity (biological characteristics)**	Inherited diseases/predisposition to disease	
	Genetic polymorphisms	
	Age (young/elderly)	
	Pregnancy/developing fetus	
	Race/ethnicity/culture	
	Mental health (coping skills)	
	Low intelligence	
	Low birth weight	

**Differential exposure (increased chemical burden)**	Old, substandard housing	Old, substandard housing
	Cleanliness/sanitation	Inadequate air flow
	Home use of pesticides	Increased air pollutant exposure
	Substandard hygiene	Traffic density
	Poor ventilation	Proximity to hazardous waste sites
		Proximity to waste disposal sites
		Proximity to industrial releases

**Differential preparedness and recovery (social environment and behavior)**	SES	SES
	Family instability	Crime and violence
	Personal nutrition	Lack of community resources
	Social support	Crowding
	Obesity	Food supply
	Smoking	Access to quality health care
	Drug addiction	Substandard schools
	Chronic underemployment	Concentration of poverty
	Other aspects of psychosocial stress	Racial segregation
	Health care access	Noise
	Health behaviors	Civil engagement/political empowerment
	Reproductive events	Social capital

Sources: [2,26,39,40].

**Table 3.** Indicators in current tools to assess potential cumulative risk in communities.

**Draft CalEPA Cumulative Impacts Assessment [2]**	**NJDEP Preliminary Screening Method to Estimate Cumulative Environmental Impacts [5]**	**NJDEP Strategies for Addressing Cumulative Impacts in Environmental Justice Communities [3]**	**US EPA’s NJSEAT [75]**
**Measures of Sensitive Population and Social Indicators**			

**Sensitive Populations**	**None**	**Social Determinants**	**Social Demographic Indicators**

% of population under age 5		Age of housing	% of population living in poverty
% of population over age 65		Proportion of population who are children	% of population counted as minority
**SES**		Proportion of population over age 60	% of population 25 years old and
% Non-white residents		Poverty rate	over without a high school diploma
Median household income		Median family income	% of population over 65 years of age
% of residents living below 2X		Racial and ethnic composition of population	% of population under 5 years of age
National Poverty Level		Unemployment rate	% of population of limited English proficiency
		Some measure of parks/recreational space	

**Measures of Environmental Exposure Burden**			

**Exposures**	**Exposures**	**Pollution burden**	**Environmental indictors**

PM_2.5_ concentrations	NATA cancer risk	Lead in blood of children age 6 or younger	NATA cancer risk
Ozone concentrations	NATA diesel exposure	RCRA sites	NATA non-cancer risk
Releases from industrial facilities (TRI data)	Estimated benzene emissions	TRI	NATA non-cancer diesel PM
	Traffic (all)	US EPA National Priorities List sites	Toxic chemical emissions and transfers from industrial facilities
	Traffic (trucks)	Power plants	
	Density of major regulated sites	Treatment, storage, and disposal facilities	Population-weighted ozone monitoring data
	Density of known contaminated sites	Brownfields	
	Density of dry cleaners	Known contaminated sites	Population-weighted PM_2.5_ monitoring data
	Density of junkyards	Municipal incinerators	
		Resource recovery landfills	
		Incinerator ash landfills	
		Dry cleaners	
		Sewage treatment plants	
		Gasoline stations	
		Municipal solid waste landfills	
		Trash transfer stations	

**Environmental effects**			**Compliance indicators**

Hazardous waste and cleanup sites			Inspections of major facilities
Leaking underground fuel tanks			Violations at major facilities
			Formal actions at major facilities
			Facility density based on all facilities in US EPA’s facility registry system

**Measures of Existing Public Health Problems**			

**Public health**		**Existing health problems**	**Human health indictors**

Low birth weight		Total cancer incidence rate	% infant mortality
Cancer mortality rate		Total cancer death date	% low birth weight births
Asthma hospitalization rate		Asthma: hospitalization rate	
		Asthma: emergency department visits	
		Chronic lower respiratory disease	
		Carbon monoxide poisonings	
		All-cause mortality rate	
		Coronary heart disease rate	
		Low birth weight rate	
		Infant mortality rate	
		Birth defect rate	
		Some measure of violence/crime	

		**Other**	

		**Availability of preventive services**	
		Childhood lead screening rate	
		Other?	
		**Basic information**	
		Total population of census tract	
		Size (area) of census tract	

NATA = National Air Toxics Assessments; RCRA = Resource Conservation and Recovery Act; TRI = Toxic Release Inventory.

**Table 4.** Studies of effect modification of social stresses on chemical health impacts.

**Health outcome**	**Chemical stressor**	**Non-chemical stressor**	**Results**	**Reference**
**Mortality**				
***Short-term studies***	CoH (PM indicator); SO_2_	SES indicators: unemployment, poverty, education, high manufacturing employment	Effect modification by SES measures; slightly higher relative risks and more significant results across the lag periods tested	[89]
	PM_2.5_	SES indicators: household income, poverty, education	Effect modification only significant for household income	[90]
	PM_10_ adjusted for O_3_, SO_2_, NO_2_, CO	SES indicators: education, annual income	No effect modification by SES	[91]
	PM_10_	SES indicator: education	Evidence of weak effect modification by education	[87]
	PM_10_	SES indicators: unemployment, poverty level, education	No effect modification by SES	[92]
	PM_10_, O_3_	SES indicator: sociospatial development index (based on homes with electricity, homes with piped water and drainage, literacy, and indigenous language speakers)	PM_10_ not associated with mortality; ozone was significantly associated with mortality, but no consistent effect modification observed	[93]
	PM_10_	SES indicators: education, income, living in slums	Effect of PM on respiratory mortality was negatively correlated with % college education, % family income > $3,500, living in slums	[94]
	PM_10_	SES indicator: composite index	Larger effect in higher SES areas but not statistically significant	[95]
	TSP, CO, NO_2_, SO_2_, O_3_, PM_10_, CoH, PM_10–2.5_	SES indicator: income	Only NO_2_ was associated with mortality in low income groups	[96]
***Short-term studies***	PM_10_	SES indicator: education	Larger mortality risk estimates were observed in least-educated for all cause, respiratory, and heart disease-related mortality	[97]
	PM_10_	SES indicator: income, index that includes education, occupation, unemployment rate, family size, crowding, home ownership	The PM_10_-mortality association was greater in lower income and lower SES communities	[98]
	O_3_	SES indicator: education, income, unemployment, poverty	Effect modification only for unemployment; higher mortality rates for higher unemployment	[99]
***Long-term studies***	PM_2.5_, sulfates	SES indicator: education	Significant effects for both PM_2.5_ and sulfates in least educated	[84]
	PM_2.5_, sulfates	SES indicator: education	Patterns are similar to previous study but effect modification is less clear; for ischemic heart disease pattern was reverse (most educated has greatest risk)	[85]
	TSP, BS, NO_2_	SES indicator: education	No effect modification by educational attainment	[100]
	BS	SES indicator: education	No effect modification by educational attainment	[101]
	TSP, SO_2_	SES indicator: income	Relative risks were higher for the low household income category	[102]
	Air pollution index: sum of standardized measures of TSP and SO_2_	Deprivation index (includes unemployment and education)	No effect modification	[103]
**Neurological effects**	Blood lead	SES indicator: income	Cognitive deficits (Kaufman Assessment Battery) associated with neonatal blood lead only in poorer families	[104]
	Blood lead	SES indicator: income	No modifying effect of SES on blood lead-learning/IQ association, but observed trend of greater vulnerability in lower SES subgroup	[105]
	Blood lead	SES indicator: parents’ occupational prestige	Modifying effects by SES were observed for IQ and blood lead, but interaction became non significant when adjusted for other factors (age at testing, iron status, birth weight, *etc.*)	[106]
	Blood lead	SES indicator: composite index including education and father’s occupation	Effect modification of lead-related decreased performance in visual-motor integration and choice reaction tests	[107]
	Blood lead	SES indicators: Hollingshead’s Four-Factor Index of Social Class, a measure of parents’ occupational and educational achievements	Modifying effects by SES were observed for Mental Development Index and blood lead only at ages 18 to 24 months	[108]
	Bone lead	SES indicators: neighborhood psychosocial hazards (neighborhood violent crimes, 911 calls, *etc.*)	Psychosocial stress exacerbated effects of lead on 3 of 7 cognitive measures	[109]
**Cardiovascular Disease**	Lead (bone lead)	SES indicators: stress (based on standardized questionnaire and self-reported)	Effects of lead on hypertension were more pronounced in stressed individuals	[110]
	O_3_, CO, NO_2_	SES indicators: education, income	No effect modification by SES on cardiac hospital admissions	[111]
**Asthma and other respiratory diseases**	NO_2_ (proxy for traffic)	SES indicators: exposure to violence	Elevated risk of developing asthma with increased NO_2_ exposure only in children with higher exposure to violence	[69]
	Traffic-related air pollution (Nitrogen Oxides)	SES indicators: parental education, parental stress	High parental stress was associated with higher incidence of traffic-related risk of developing asthma. An increased risk of asthma was also observed for low SES families exposed to air pollution	[112]
	O_3,_ SO_2_, NO_2_	SES indicators: education, income	Greater hospitalizations for respiratory effects in lower education and lower income strata	[113]
	NO_2_, SO_2_, O_3_, CO	SES indicator: average household income adjusted for household size	Male children had higher asthma hospitalizations in low SES group with exposure to NO_2_; female children had higher asthma hospitalizations for SO_2_ in the low-income group. No associations for O_3_ or CO	[114]
	NO_2_	SES indicator: insurance status	Children without insurance had higher risk of asthma admissions than those with private insurance	[115]
	PM_10_, O_3_, sulfates, strong acidity	SES indicator: insurance status	The overall hospital admissions association for both O_3_ and PM_10_ was driven by the uninsured minority population	[116]
	NO_2_	Life stress	Greater inflammatory markers associated with high stress in low pollution exposure group	[117]

CoH = coefficient of haze; SO_2_ = sulfur dioxide; PM_10_ = particulate matter > 10 μm; O_3_ = ozone; NO_2_ = nitrogen dioxide; CO = carbon monoxide; TSP = total suspended particulate matter
